# Nanoparticle Strategies to Improve the Delivery of Anticancer Drugs across the Blood–Brain Barrier to Treat Brain Tumors

**DOI:** 10.3390/pharmaceutics15071804

**Published:** 2023-06-23

**Authors:** Wouter J. F. Vanbilloen, Julian S. Rechberger, Jacob B. Anderson, Leo F. Nonnenbroich, Liang Zhang, David J. Daniels

**Affiliations:** 1 Department of Neurologic Surgery, Mayo Clinic, Rochester, MN 55905, USArechberger.julian@mayo.edu (J.S.R.);; 2 Department of Neurology, Elisabeth-Tweesteden Hospital, 5022 GC Tilburg, The Netherlands; 3 Department of Molecular Pharmacology and Experimental Therapeutics, Mayo Clinic, Rochester, MN 55905, USA; 4 Medical Scientist Training Program, Mayo Clinic College of Medicine and Science, Rochester, MN 55905, USA; 5 Hopp Children’s Cancer Center Heidelberg (KiTZ), 69120 Heidelberg, Germany; 6 Clinical Cooperation Unit Pediatric Oncology, German Cancer Research Center (DKFZ) and German Consortium for Translational Cancer Research (DKTK), 69120 Heidelberg, Germany

**Keywords:** nanoparticle, liposome, extracellular vesicle, chemotherapy, targeted therapy, drug delivery, blood–brain barrier, brain tumor, glioma

## Abstract

Primary brain and central nervous system (CNS) tumors are a diverse group of neoplasms that occur within the brain and spinal cord. Although significant advances in our understanding of the intricate biological underpinnings of CNS neoplasm tumorigenesis and progression have been made, the translation of these discoveries into effective therapies has been stymied by the unique challenges presented by these tumors’ exquisitely sensitive location and the body’s own defense mechanisms (e.g., the brain–CSF barrier and blood–brain barrier), which normally protect the CNS from toxic insult. These barriers effectively prevent the delivery of therapeutics to the site of disease. To overcome these obstacles, new methods for therapeutic delivery are being developed, with one such approach being the utilization of nanoparticles. Here, we will cover the current state of the field with a particular focus on the challenges posed by the BBB, the different nanoparticle classes which are under development for targeted CNS tumor therapeutics delivery, and strategies which have been developed to bypass the BBB and enable effective therapeutics delivery to the site of disease.

## 1. Introduction

### 1.1. Primary Brain and Other Central Nervous System Tumors

Primary brain and other central nervous system (CNS) tumors are a diverse group of neoplasms that occur within the brain and spinal cord. These tumors can arise from various cell types, including glial cells, neurons, meningothelial cells, and embryonic cells. In adults, brain tumors account for approximately 2% of all cancer diagnoses and 3% of deaths due to cancer [1]. It is estimated that 700,000 people in the U.S. are living with a primary brain tumor, and approximately 90,000 more will be diagnosed in 2023 [2]. More than two-thirds of patients diagnosed with glioblastoma (GBM), the most aggressive type of brain cancer in adults, will succumb to their disease within 2 years of diagnosis, and an estimated 20,000 adults in the U.S. die from primary cancerous brain tumors each year [1,3]. In individuals under the age of 20, brain tumors are the second most common category of cancer and the leading cause of cancer-related death [4,5]. In children, H3 K27-altered diffuse midline glioma (DMG) is the most lethal form of brain cancer, associated with an abysmal prognosis and a 5-year survival rate of less than 2% [4,6]. Additionally, children diagnosed with a brain tumor who survive and enter adulthood will often be affected by the long-term consequences of exposing the developing brain to medical interventions [7]. Overall, brain tumors remain a significant source of morbidity and mortality for which diagnosis and treatment require extensive resource allocation and sophisticated diagnostic and therapeutic technology [8].

Treatment options for brain tumors depend on the type, location, and stage of the tumor, as well as the patient’s age and overall health [9]. Most brain tumors have proved challenging to treat, due in large part to the molecular features of these tumors, which frequently work in concert to impede advancements in therapy [10]. Surgical resection, chemotherapy, and radiation therapy (RT) remain the primary treatment modalities [11,12]. Given the lack of durable therapies for most brain tumors, there is a dire unmet gap in clinical practice for improved therapeutic modalities based on the unique molecular underpinnings of individual tumors.

As our understanding of the intricate biology that mediates tumorigenesis and progression increases, the integration of molecularly targeted agents, which can target key factors on tumor cells, the tumor microenvironment, or the patient’s immune system, into conventional therapeutic regimens may provide a substantial benefit for patients with otherwise incurable brain tumors [12,13,14,15]. However, a multitude of factors, such as molecular heterogeneity, invasion of tumor cells outside the bulk tumor core identified on imaging, as well as the brain–CSF barrier and blood–brain barrier (BBB), which prevent the buildup of xenobiotics within the CNS, may limit the efficacy of these promising therapeutic strategies [16].

### 1.2. Blood–Brain Barrier

Although progress has been made in identifying potentially targetable vulnerabilities for the treatment of brain tumors, crossing the BBB and achieving therapeutic drug levels at the tumor remain significant obstacles. The BBB is an anatomical and biochemical barrier that works by tightly controlling the permeation of ions, macromolecules, and nutrients into the brain in order safeguard it from potentially harmful substances like toxins, pathogens, and drugs present in systemic circulation [17,18]. This is accomplished with cooperative work by multiple cellular components, including brain capillary endothelial cells (ECs), pericytes, and astrocytic glia cells, which orchestrate a complex intra- and intercellular barrier network [19,20,21]. Together, these cells not only serve a structural purpose, but they also function as a neurovascular unit that regulates BBB integrity and affects drug penetration into the brain [22,23].

Unlike the peripheral microvasculature, ECs located at the BBB are characterized by having only few fenestrations and pinocytic vesicles and are tightly linked by tight junctions (zonulae occludentes), which together act as a physical barrier, limiting the unrestricted diffusion of substances from the bloodstream into the brain [24,25]. Claudins, occludins, and junctional adhesion molecules (JAM-A, -B, and -C) are among the most abundant proteins that make up the zonula occludens complex for restricting paracellular transport [26,27]. Molecules that cannot diffuse easily across lipid bilayers, such as small hydrophilic drugs and therapeutic macromolecules, including antibodies and antibody–drug conjugates, therefore, cannot normally accumulate in meaningful amounts due to this physical barrier [28].

Polar nutrients like some amino acids, hormones, carbohydrates, and vitamins are transported across the BBB through carrier-mediated influx transporters such as the L-type amino acid transporter 1 (LAT1), glucose transporter 1 (GLUT1), and organic anion transporter polypeptides (OATPs) [29]. Similarly, large molecules like insulin, transferrin, and some vitamins can be shuttled into the brain by multiple transport mechanisms, including receptor-mediated endocytosis and different transcytosis pathways [17,30].

Efflux transporter proteins found on the luminal and abluminal side of the EC membrane effectively transport many lipophilic molecules through the luminal EC membrane back into the capillary lumen [31]. Many small molecules including drugs that can otherwise readily diffuse across plasma membranes have substrate properties for these efflux pumps [32]. ATP-binding cassette (ABC) family members, such as P-glycoprotein (P-gp), breast cancer resistance protein (BCRP) and multidrug resistance-associated protein 1 (MRP1), have been studied in detail and reported to limit brain distribution of numerous anticancer drugs [33,34,35,36,37]. Therefore, the physical and biochemical characteristics of the BBB greatly restrict the delivery of therapeutic agents to the brain, which may reduce the effectiveness of many systemically administered therapies [38,39,40].

The integrity of the BBB within the tumor area can vary depending on the particular tumor type and is referred to as the blood–tumor barrier (BTB) by many [18,40,41]. While in the majority of brain tumor patients, the BBB is disrupted to some extent, its integrity has been shown to be variable or remain intact using dynamic contrast enhanced magnetic resonance imaging (MRI), especially in the peritumoral regions [42,43,44]. This is particularly true for children with DMG and some medulloblastoma subtypes (e.g., sonic hedgehog (SHH) activated tumors), where little or no contrast enhancement on MRI indicates a largely intact BBB [45]. Furthermore, the structure of the BBB and the expression pattern of efflux transporters has been shown to vary in different patient populations [46,47]. Based on age, brain location, and efflux transporter type, a distinct maturation profile was reported in brain cortical and ventricular tissue of more than 50 human patients, including fetuses, newborns, children, and adults [48]. These findings imply that major advancements in the treatment of brain tumors will require the delivery of therapeutic agents across the BBB to all tumor regions regardless of individual patient and tumor characteristics.

### 1.3. Nanoparticle Strategies in Neuro-Oncology

Nanoparticles (NP) are a diverse group of nanoscale objects characterized by their size—usually ranging from 1 to 100 nm—which have gained attention as drug delivery systems to improve the biodistribution of therapeutic agents through improved solubility and stability, ability to cross biological barriers, and organ- or cell-specific targeting in order to either increase efficacy, reduce side effects, or both [49,50]. Several NP-based drug formulations have been approved by the U.S. Food and Drug Administration (FDA) in other oncology fields, yet no successful clinical trials have been conducted in brain tumors, highlighting an important translational gap [51]. While there exists an abundance of promising preclinical studies, the clinical failure of nanoparticle formulations in brain tumors to date is likely related to an incomplete reflection of the BBB and other anatomical and physiological hurdles that must be surmounted to obtain access to this highly protected tumor environment. In this review, we will provide an update on and highlight recent developments in NP-based drug delivery systems across the BBB, with a specific focus on the therapeutic application for brain tumors, along with existing constraints and possible future paths to overcome translational limitations (Table 1) [52,53,54,55,56,57].

## 2. Nanoparticle Classes under Investigation as Drug Delivery Systems for Brain Tumors

**Figure 1:**
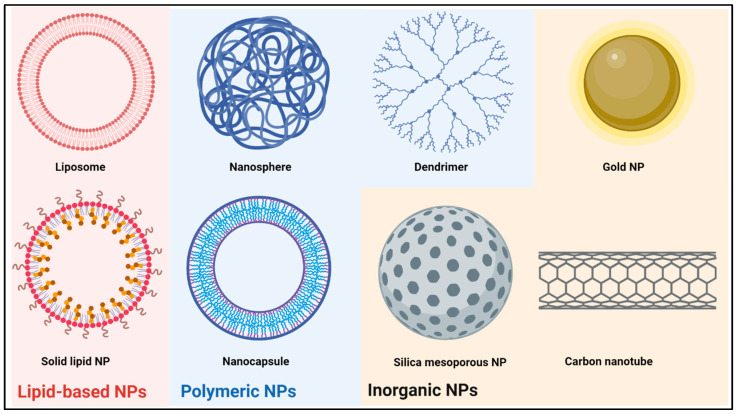
General structure of the most common synthetic nanoparticles (NPs) used for drug delivery. Created with BioRender.com.

Several classes of NPs are being pursued for the development of CNS-targeted drug delivery systems. While many paradigms are applicable across different NP categories, important differences are to be observed. Synthetic (Figure 1) and biological NPs make up the two major categories of NPs. The former are characterized by a high degree of control over pertinent physicochemical properties, such as size and surface charge, and include lipid-based NPs, polymeric NPs, and inorganic NPs, among others. Biological NPs are either fully derived from living cells or at least partly constructed through a biological process, offering biocompatibility through their intrinsically functionalized membranes while foregoing some of tunability of synthetic NPs. Although the various NP classes have been thoroughly reviewed elsewhere, we will briefly discuss the main categories that have been investigated for the application in brain tumor therapy in the following section [58,64,69,72,82,83,84,85,86,87].

### 2.1. Lipid-Based Nanoparticles

The two main lipid-based NPs are liposomes and solid lipid NPs (SLNs). Liposomes are spherical vesicles consisting of at least one phospholipid bilayer around an aqueous core, typically ranging from 30 to 2500 nm in size [88]. Despite the fact that several liposomal drug formulations are approved as systemic drug delivery system by the FDA, none are currently in clinical use for the treatment of brain tumors [51]. The main advantage of liposomes is the easy manufacturing process, allowing for the modulation of physicochemical properties and phospholipid composition. By introducing a double lipid bilayer or by encasing several vesicles inside a second membrane, multilaminar or multivesicular liposomes can be produced depending on the phospholipid makeup [58]. Furthermore, surface modifications using proteins, peptides or polymers are used to alter systemic circulation time and allow for targeted delivery [59,60]. A broad range of therapeutics, including both lipophilic and hydrophilic drugs, can be encapsulated in the lipid bilayer or the aqueous core, expanding the use of liposomal drug carriers [58]. An important limitation for clinical use is the low bioavailability due to efficient phagocytosis by the reticuloendothelial system (RES), resulting in preferential accumulation in the liver and spleen [61,89]. Moreover, even though liposomes are regarded as highly biocompatible, complement activation-related pseudoallergy (CARPA) is a common adverse reaction, occurring in 25–45% of patients upon first administration [62,90].

Solid lipid NPs (SLNs) are another common subset of lipid-based NPs. They differ from liposomes in that they are built from a phospholipid monolayer around a lipophilic core matrix. Within this core, micellar structures can be formed around hydrophilic cargo. SLNs have been mainly used as drug delivery systems for nucleic acids [63,91]. Ionizable phospholipids with near-neutral charge in physiologic pH form micelles around nucleic acids, while in acidic endosomes, these phospholipids become charged, promoting endosomal escape [63,91]. Combined with their simple synthesis and good biocompatibility, SLN are a promising drug delivery system for brain tumor therapy, and small molecule drugs have been successfully delivered to the brain using SLN [92,93,94]. However, as with liposomes, rapid accumulation in the RES is a major limiting factor [64,94].

Besides liposomes and SLNs, nanoemulsions have also been considered to improve drug delivery to CNS tumors. Nanoemulsions are colloidal suspensions, usually consisting of nanosized lipid droplets in aqueous media stabilized by surfactants [95]. They have gained attention due to their ability to cross biological barriers, increase bioavailability of hydrophobic therapeutics, ease of manufacturing, stability, and biocompatibility [95,96,97,98]. However, although some groups have been able to attach targeting ligands, the potential for modification is more restricted compared to other NPs [97]. While oral and intravenous administration have been investigated, the intranasal delivery of nanoemulsions was the most effective in the treatment of CNS tumors in animal models [99,100,101].

### 2.2. Polymeric Nanoparticles

Polymeric NPs are a diverse group of synthetic NPs. They are built using a natural or synthetic core polymer that either forms a solid nanosphere or a liposome-like nanocapsule, in which the core polymer forms a shell around a usually aqueous core. Most frequently used polymers in neuro-oncology research are poly (lactic-co-glycolic acid) (PLGA), poly (β-amino ester), polystyrene (PS), polyanhydride, chitosan, and polycaprolactone. Through the inclusion of various co-polymers, polymeric NPs exhibit a high potential for modifying stability and surface charge, enabling drug release timing to be altered from days to weeks [65,102,103,104,105]. As with liposomes, further surface functionalization is possible [66,67,106]. Drugs can either be attached to the surface, embedded in the nanosphere or nanocapsule shell, or can reside in the aqueous core, enabling the delivery of both lipophilic and hydrophilic cargo with different molecular weights [69].

Dendrimers are a specific type of polymeric NP that can be distinguished from other polymeric NPs by their structural differences. They are built from an initiator core that anchors a variable number of ‘generations’ of branched layers, terminating in an outer layer of functionalized surface groups that can harbor imaging, targeting or therapeutic moieties. Sizes typically range from 1 to 15 nm, growing 1–2 nm with each generation while doubling the amount of surface groups, allowing a high degree of control over size and surface chemistry [68]. Commonly used dendrimers to target the CNS are polamidoamine (PAMAM) and dendrigraft poly-L-lysine (DGL). Small molecule drugs and nucleic acids are the most frequent payloads, although a wide variety of therapeutics can be attached to the outer branches and encapsulated in the inner void spaces [84].

Overall, polymeric NPs are excellent candidates for drug delivery because they are biodegradable into nontoxic components, highly tunable, and several polymers have been FDA-approved for clinical use as either systemic or topical drug delivery system [51,107,108,109,110,111,112,113]. However, the low drug loading capacity of most polymeric NPs and rapid clearance by the RES are limiting factors [70,71]. Notwithstanding these limitations, several clinical trials of polymeric NPs for systemic drug delivery in cancer are ongoing, although none of which target CNS neoplasms [114].

### 2.3. Inorganic Nanoparticles

Inorganic NPs are synthesized from inorganic compounds, such as gold, silica, iron and carbon, and can be manufactured in a wide array of sizes and shapes. Gold NP (AuNP), for example, form nanospheres, nanorods, nanoflowers, nanoshells and nanocages [72]. Carbon forms quantum dots, fullerenes or nanotubes, and silica is usually used to make mesoporous NPs (MSN) [85,86,115]. Different inorganic NPs have unique properties, such as the photothermal properties of gold or the magnetic properties of iron NPs, giving rise to other uses such as photothermal radiosensitization therapies or imaging applications, respectively [73,74]. AuNPs, carbon nanotubes and mesoporous silica NPs in particular have been explored as drug delivery systems. AuNPs have the most diverse applications, providing a high surface-to-volume ratio and being able to conjugate a wide arrange of small molecules, proteins or nucleic acids directly to the surface [72]. MSN provide a large surface area, can be modulated to harbor different pore sizes fitting various types of drugs, and allow a high degree of control over drug release [87]. Carbon nanotubes can be loaded with hydrophobic drugs, and the surface decorated with various therapeutic and targeting moieties [116]. The main disadvantages of inorganic NPs are toxicity concerns and low solubility leading to aggregation [74,75]. While AuNPs are generally regarded as safe, MSNs are prone to causing hemolysis through interaction with the red blood cell plasma membrane, and especially prolonged exposure to carbon nanotubes induces cytotoxicity in vitro and lung and liver toxicity in rodents [76,87].

### 2.4. Biological Nanoparticles

**Figure 2:**
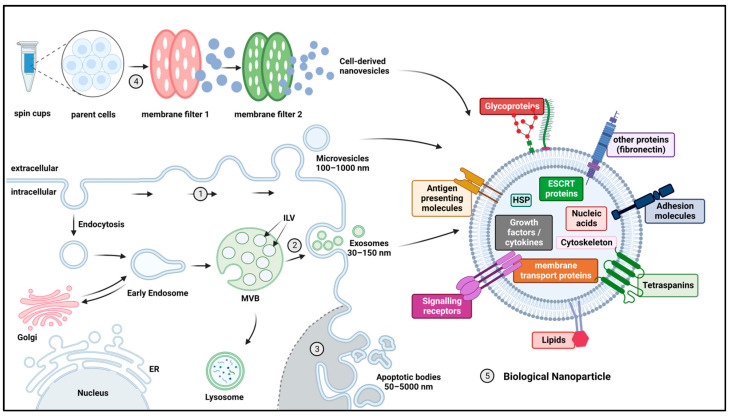
Biogenesis, production and general structure of biological NPs. (1–3) Extracellular vesicles (EVs) are differentiated into three groups based on their biogenesis (**1**) Microvesicles are small to medium sized vesicles (100–1000 nm) that originate from outward budding of the plasma membrane (PM), incorporating cytosolic proteins. (**2**) Exosomes are small, homogenous vesicles (30–150 nm), formed by inward budding of the endosomal membrane, forming intraluminal vesicles (ILVs) in an MVB and subsequently transported to either the PM, where they are released as exosomes, or to the lysosome for degradation. (**3**) Apoptotic bodies are usually large (50–5000 nm), heterogeneously shaped vesicles, shed by cells undergoing apoptosis. (**4**) Cell-derived nanovesicles (CDNs) are generated through mechanical extrusion, ultrasonication or freeze–thawing of parent cells. (**5**) EVs and CDN are both constructed from a phospholipid bilayer, inherently functionalized with various groups of membrane proteins. While some proteins are more common in certain vesicle types, there is considerable overlap. In the lumen, a diverse range of cargo proteins and nucleic acids can be identified. Abbreviations: MVB = multivesicular body, ER = endoplasmic reticulum, HSP = heat shock protein, ESCRT = endosomal sorting complexes required for transport. Created with BioRender.com.

Biological NPs (Figure 2) mainly encompass extracellular vesicles (EVs) and cell-derived nanovesicles (CDN). Extracellular vesicles (EVs) are a group of naturally occurring NPs with a phospholipid bilayer membrane that are produced by most cells studied to date [117,118,119,120,121,122,123,124,125,126,127,128,129]. Based on their biogenesis, EVs are classified into three main groups: exosomes, microvesicles and apoptotic bodies. Exosomes have attracted the most attention as a drug delivery mechanism, but it can be difficult to distinguish them from other EVs because of the overlap in size and biological make-up [130,131,132,133]. Therefore, in accordance with the MISEV2018 consensus paper, we will use the term EV in the rest of this review [133].

Contrary to earlier theories that EVs were primarily responsible for the removal of unwanted proteins from cells, they have been demonstrated to play a significant role in intercellular communication in both physiological and pathological processes [77,134,135,136,137,138,139,140,141]. The strict regulation of EV lipid bilayer composition, which is different from that of the parent cell, as well as the selective inclusion/exclusion of certain membrane and intra-vesicular proteins that are present in the parent cell, are indications of this biological function [142,143,144,145,146]. While some proteins are related to their biogenesis, others are important for their biological function and differ between EVs from different parent cells, e.g., combinations of α- and β-chains of integrins changing their organotropism, or the presence of MHC molecules in EVs from dendritic cells [77,147,148]. Nucleic acids relevant to their function are also regularly identified as a cargo of EVs [149]. Although the inherently functionalized membrane provides high biocompatibility and some degree of organotropism, further surface modifications have been applied in an effort to improve drug delivery [77,150,151,152]. Therapeutics can be introduced into EVs either before harvesting (through the overexpression of desired proteins or nucleic acids in engineered parent cells) or after harvesting (through electroporation, sonication or other loading methods) [78]. EVs have shown little to no inherent toxicity in previous in vivo studies [77,79,80,129,153]. As with other NPs, however, a large portion of EVs are captured in the RES [61,81,154]. Furthermore, harvesting and purifying EV in sufficient quantities for research or clinical purposes are time consuming and complex, limiting their application at present [78].

Cell-derived nanovesicles (CDNs)—in contrast to EVs, which are created through a tightly regulated biological process—are produced through mechanical extrusion, ultrasonication, or the freeze–thawing of parent cells [155,156]. These techniques cause donor cells to release CDN in high quantities, dramatically increasing production yield compared to EVs, while preserving biological properties [155,157]. There is a substantial overlap in membrane proteins and smRNA contents between EV and CDN, although studies have demonstrated a difference in membrane lipid composition [155,156]. The in vitro and in vivo behavior of CDNs as well as the achievable drug loading capacity are also similar to EVs [155,158]. Overall, preliminary findings imply that CDNs might offer a useful EV substitute by combining the benefits of EVs with significantly improved production scalability.

## 3. Engineered Nanoparticles to Enhance Targeted Drug Delivery to CNS Tumors

**Figure 3:**
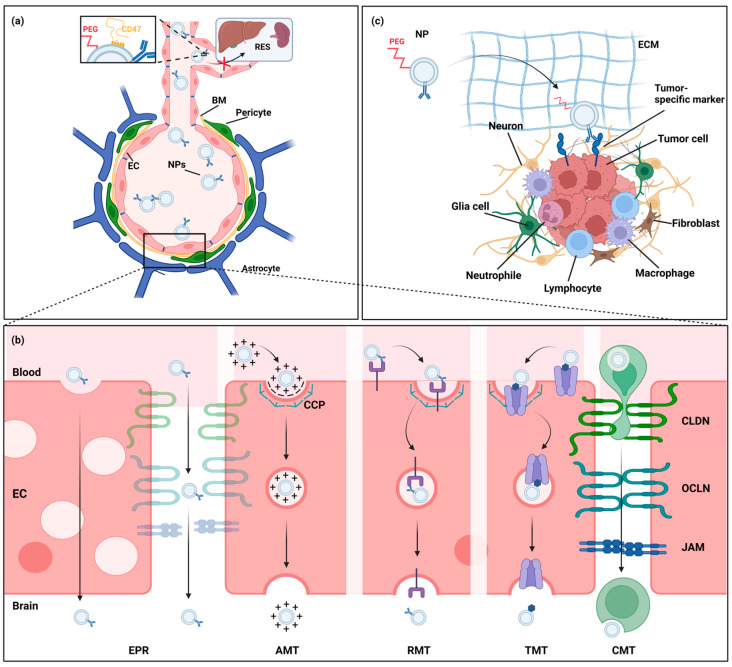
Paradigms of NP-mediated delivery to the central nervous system (CNS). (**a**) The majority of systemically administered NPs are subject to rapid clearance by the reticuloendothelial system (RES). Two important strategies to avoid recognition by macrophages and inhibit phagocytosis are PEGylation or CD47 expression, respectively. (**b**) While the blood–brain barrier (BBB) considerably impairs drug delivery to the CNS, NP-mediated drug delivery can exploit various biological transport pathways to overcome this limitation. CNS neoplasm-induced neoangiogenesis gives rise to blood vessels with an immature BBB, marked by leaky tight junctions and fenestrated endothelial cells (ECs), allowing NPs to take advantage of the enhanced permeability and retention (EPR) effect. Adsorption-mediated transcytosis (AMT), receptor-mediated transcytosis (RMT), and transporter-mediated transcytosis (TMT) are forms of endosomal transport, triggered by electrostatic interactions, ligand–receptor interactions or substrate-transporter interactions, respectively, that can be leveraged by targeted NPs. Furthermore, in cell-mediated transport (CMT), NPs have been loaded into mesenchymal stem cells (MSC) and white blood cells (WBC) that migrate over the BBB in response to tissue damage and inflammation. (**c**) PEGylation allows for improved migration through the extracellular matrix (ECM), while tumor-specific ligand conjugation increases NP targeting capabilities. Abbreviations: PEG = polyethylene glycol, BM = basal membrane, CCP = clathrin-coated pit, CLDN = claudin, OCLN = occluding, JAM = junctional adhesion molecule. Created with BioRender.com.

Despite the abundance of available NP formulations, the majority of NPs are unable to efficiently reach the CNS, necessitating the development of advanced NP designs for brain tumor purposes that take into account the entire delivery cascade [159]. While BBB penetrance is the most widely acknowledged prerequisite, attaining adequate, persistent plasma concentrations; having the ability to migrate the extracellular matrix of the brain parenchyma; and being able to selectively deliver therapeutic payloads to tumor cells are equally important for achieving a therapeutic effect (e.g., CRITID procedure of brain-targeting drug delivery) [54]. In this section, we will review the various strategies that have been applied across NP classes to address these biological barriers in the treatment of brain tumors (Figure 3) [54,160,161].

### 3.1. Nanoparticle Clearance and Blood Circulation Time

Achieving adequate and persistent plasma concentrations is crucial for systemically administered drugs to achieve and maintain effective CNS concentrations in order to impart its therapeutic effect. As mentioned earlier in this review, most NPs are rapidly captured in the bloodstream by the RES. While this has been long known for liposomes and polymeric NPs, EVs show a similar clearance pattern despite their biological origin, with half lives of less than ten minutes and significant accumulation in the liver, spleen and lungs [61,71,81,89,154,162]. PEGylation is the most common modification to improve NP circulation time, but this has been shown to decrease the capacity for cellular interaction [60,71,163,164,165]. While the improved pharmacokinetics of PEGylated NPs have been shown to enhance CNS delivery in some scenarios, a detrimental effect on BBB crossing has been reported in others [166,167]. Furthermore, while PEG is classified by the FDA as generally regarded as safe (GRAS), production of anti-PEG antibodies has been detected after repeated dosing of PEGylated NP, resulting in accelerated blood clearance (ABC) known as the ABC effect [90,168].

A more recently explored alternative strategy is the expression of CD47, a ligand of signal-regulatory protein alpha (SIRPα) on phagocytes, inhibiting phagocytosis, as a natural ‘don’t eat me’ signal [169]. Kamerkar et al. showed reduced clearance of EVs and liposomes after increasing CD47 expression [152]. This strategy has been further leveraged by Belhadj et al. into a combined ‘eat me/don’t eat me’ strategy, which consists of first administering decoy EVs to saturate the RES, followed by CD47-expressing drug-loaded EVs [170]. Using this strategy, the authors reported increased tumor accumulation of drug-loaded EVs and improved survival rates in a lung cancer mouse model. This strategy is also applicable to other NPs and has been shown to be superior to PEGylation by some studies [171,172].

Additionally, physicochemical properties such as NP size and surface charge impact systemic circulation time [173,174,175]. NPs smaller than 5 nm are rapidly excreted through renal glomerular filtration [176]. Zhang et al., markedly reduced renal clearance of a PAMAM dendrimer by slightly increasing the size from 4.3 nm to 6.7 nm [176]. Conversely, NPs larger than 200 nm are more likely to be captured by the RES [177]. Furthermore, in phospholipid-based NPs, lipid composition can also influence clearance rates [178,179,180]. More recently, the effect of different NP shapes has gained considerable interest, as it has been demonstrated that rod-shaped NPs interact with cells less frequently, leading to decreased clearance by the RES [181,182,183].

### 3.2. Nanoparticle Strategies to Enhance Drug Delivery Past the BBB

The inability to cross the BBB and achieve therapeutic concentrations is a significant drawback of most conventional drugs [19]. While unaltered NPs exhibit some degree of BBB penetrance, engineered NPs have been developed to improve drug delivery over the BBB. These formulations exploit biological processes such as endogenous transport pathways or the migration of mesenchymal stem cells (MSC) and white blood cells (WBC) in response to tissue damage or inflammation [128,132,184,185,186,187,188,189,190]. Other strategies for bypassing the BBB altogether, such as intranasal delivery, convection-enhanced delivery (CED), or temporary BBB disruption, are also being investigated in combination with NPs.

#### 3.2.1. Nanoparticle Modifications to Increase BBB Passage

Although their physicochemical properties largely prevent most NPs from crossing the BBB, they can either inherently or after surface modification take advantage of natural transcytosis pathways. Transcytosis is a form of active vesicular transport, initiated by endocytosis from the luminal side of ECs, from where endosomes are sorted to be degraded in lysosomes, returned to the bloodstream, or transported to the abluminal side of the EC. In brain capillaries, endocytosis is primarily mediated by clathrin-coated pits (CPs) [191]. Three pathways are distinguished based on the trigger for endocytosis: adsorption-mediated transcytosis (AMT), receptor-mediated transcytosis (RMT) and transporter-mediated transcytosis (TMT). Although an in-depth analysis of these pathways is beyond the scope of this review, we will provide a summary of the most common strategies. We kindly refer to reviews from Azarmi et al. and Moura et al. for a more comprehensive overview [192,193].

In AMT, endocytosis is initiated after the electrostatic adsorption of cationic particles to the anionic CPs. While cationic NPs, such as chitosan NPs and certain polyamidoamine (PAMAM) dendrimers, and NPs functionalized with cationic molecules have been shown to cross the BBB, AMT intrinsically lacks CNS specificity as negatively charged membranes are virtually universal to all living cells [185,194,195,196]. In contrast, RMT is a specific process, triggered by binding an EC surface receptor. Through the conjugation of either endogenous or engineered ligands for receptors predominantly expressed on brain ECs onto NPs, CNS-specific delivery of NP-encapsulated drugs can be achieved. Commonly targeted receptors include transferrin (TfR), lactoferrin (LfR), insulin, and low-density lipoprotein (LDLR) receptors as well as LDLR-related peptides (LRPs) [197,198,199,200,201,202,203]. Some authors further reported the expression of the nicotinic acetylcholine receptor (nAchR) on NPs to harness RMT [204]. Due to their potential as a dual target, being widely expressed in both tumor cells and brain EC, some receptors, such as the TfR, have undergone extensive research [198,205,206]. TfR ligands have been successfully conjugated to lipid-based, polymeric and inorganic NP, increasing target cell specificity in vitro while providing increased CNS uptake in vivo [206,207,208,209,210]. Using transferrin-coupled temozolomide-loaded PLGA NPs, Kuang et al. showed increased antitumor activity in a U87 orthotopic xenograft glioma mouse model [206]. Nonetheless, absolute NP uptake with RMT is usually low [211].

Similar to RMT, TMT is a specific process initiated by binding a transporter present on the EC surface. The most commonly investigated TMT transporters are GLUT1 and the glutathione transporter, both serving a dual role, being highly expressed on brain ECs and many tumor cells [203,212,213]. Critically, however, when targeting endogenous receptors and transporters important for brain homeostasis, the potential for serious adverse reactions should be considered, as ligand-coated NPs might competitively inhibit the transport of important nutrients to the CNS. To this end, a study using TfR-targeted oxaliplatin-loaded liposomes reported dose-dependent lethargy postinjection in mice [207]. Conversely, endogenous ligands might outcompete engineered NPs, decreasing the targeting efficiency.

Besides conjugation of targeting moieties, modulation of NP shape provides another strategy to optimize endocytosis. Anti-VCAM-1, anti-ICAM-1 and anti-TfR-coated PS nanorods showed increased brain accumulation compared to spherical PS NP in vitro and in vivo. Interestingly, spherical NPs associated significantly more with brain ECs than their rod-shaped counterparts, suggesting that spherical shapes increase nonspecific intercellular interactions [214,215,216]. Given that most NPs are spherical, this warrants further investigation of NPs with other shapes.

#### 3.2.2. Cell-Mediated and Cell-Mimicking Drug Delivery over the BBB

Another strategy to potentially enhance BBB passage is by loading NP into cells capable of migrating over the BBB, such as MSCs and WBCs, or coating them with cell membranes [217]. This way drug can be protected from degradation while carrier cells facilitate targeting to the tumor regions [188,189]. MSCs have been intensely investigated for cell-based therapies due to their regenerative properties and tumor-tropism, making them a prime candidate for NP-based drug delivery [190,218]. Roger et al. demonstrated the ability to load PLA NPs and SLN into MSCs without affecting their cell viability or ability to migrate towards glioma cells in vitro and in vivo in a U87MG orthotopic xenograft glioma mouse model after administration via CED [219]. Using a U251 heterotopic flank xenograft glioma mouse model, Li et al. reported prolonged retention and enhanced apoptosis after intratumoral injection of doxorubicin-loaded silica nanorattles attached to MSCs compared to both free drug and doxorubicin-loaded silica nanorattles [220]. Similarly, WBCs are capable of migrating over the BBB towards regions of tissue damage and inflammation [188]. Multiple groups demonstrated the ability of macrophages, neutrophils and T-lymphocytes to be loaded with different types of NPs [221,222,223,224,225]. Using monocytes as a carrier, Ibarra et al. showed enhanced accumulation of polymeric NPs in the tumor region of an GL261 orthotopic xenograft glioma mouse model [222]. Importantly, this xenograft model had a compromised BBB in the tumor region; therefore, none of these experiments were able to definitively prove NP passage over an intact BBB.

Rather than loading NPs into live cells, other groups have coated various NPs in specific cell membranes in order to attain similar benefits. For example, Zhang et al. have cloaked their NP in MSC membranes to improve BBB passage and tumor targeting, and Ji et al. packaged doxorubicin in platelet membranes as adjuvant therapy with neurosurgery, targeting the damaged vascular endothelium at the surgical margins [226,227]. Although further evaluation is needed, these ‘Trojan horse’-inspired strategies hold promise to optimize NP delivery.

#### 3.2.3. Bypassing the BBB

Rather than improving BBB penetrance, other strategies have focused on circumventing or (temporarily) disrupting the BBB entirely. Widely studied approaches include intranasal delivery, convection-enhanced delivery (CED), and focused ultrasound (FUS). While these techniques are also being investigated in combination with conventional drugs, beneficial effects of NP-encapsulation are being explored.

A systemic first pass effect and the BBB are avoided by intranasal delivery, which is envisioned by direct uptake via the olfactory and trigeminal neuroepithelia into the brain parenchyma. Upon intranasal administration of EV-encapsulated curcumin and JSI-124, a STAT3 inhibitor, Zhuang et al. demonstrated anti-inflammatory effects and reduced tumor growth in brain inflammation and orthotopic xenograft glioma mouse models, respectively [228]. Similarly, Sousa et al. reported improved antiangiogenesis, reduced tumor growth and reduced systemic drug exposure in a U87 orthotopic xenograft glioma mouse model after intranasal administration of a bevacizumab-loaded PLGA PNP compared to the free drug [229]. However, the translation relevance of intranasal delivery from animal models to humans is debated due to the relatively large size of the olfactory system in rodents, the highly variable administration efficiency, and the limited maximal doses [230].

CED is a neurosurgical technique that circumvents the BBB by directly infusing drugs into the brain parenchyma, encompassing the tumor site through the generation of a mechanical pressure gradient [231,232]. The use of convective kinetics facilitates the homogenous distribution of infused drugs at high local concentrations with minimal systemic toxicity [45,233]. Early-phase clinical trials of CED have established the safety and feasibility of this procedure in children and adults [234,235,236,237,238]. However, inadequate drug distribution and retention have been largely cited as the reasons for the failure of a phase III CED study performed in adult GBM [239,240,241]. Nanoparticle-encapsulated drugs were found to be retained in situ for longer than free drugs alone in prior in vivo experiments using CED of nanoparticles [242]. Zhang et al. further demonstrated the enhanced in vivo distribution of PEGylated liposomal doxorubicin compared to free doxorubicin in a tumor-naïve mouse model [243]. MTX110, a water-soluble nanoparticle formulation of panobinostat, distributed effectively in the brains of small and large animals following CED without clinical or neuropathological signs of toxicity up to an infused concentration of 30 μM and is currently undergoing clinical development [33,244]. Preliminary data from seven patients who received two 48 h MTX110 infusion pulses (30 or 60 μM) showed some encouraging signs of antitumor activity with repeated CED of MTX110 [237].

Lastly, a legion of options has been explored to improve brain–drug delivery via the temporary disruption of the BBB, including osmotically active agents such as mannitol and mechanical methods such as focused ultrasound (FUS). However, disruption of the BBB does not uniformly result in increased drug penetration into the brain, as it does not only increase influx but also facilitates rapid clearance out of the brain [245,246]. Notwithstanding, Nance et al. showed improved delivery of long-circulating PEGylated PS PNPs to the brain using MRI-guided FUS, suggesting that local and temporary BBB disruption in combination with longer circulating NP might improve the in vivo efficacy of administered therapeutics [247].

### 3.3. Nanoparticle Modifications to Increase Delivery to Brain Tumor Cells

After crossing or bypassing the BBB, the extracellular matrix (ECM) of the brain parenchyma forms another biological barrier NPs need to navigate to reach the target cell. While the ability to move throughout the ECM is inversely correlated with NP size, mechanical adhesion can severely limit the diffusion of NPs of any size [248]. Nance et al. demonstrated that uncoated PS PNPs of all sizes are immobilized by adhesion, and while densely PEGylated paclitaxel-loaded PLGA nanoparticles could readily move through the ECM, uncoated PLGA NPs could not [248]. The authors concluded that densely PEGylated NPs with a near-neutral charge and a size of <114 nm are most optimal for diffusion through the brain parenchyma after systemic administration. Building on these findings, Schneider et al. produced a PEGylated PS PNP decorated with a ITEM4 monoclonal antibody targeting fibroblast growth factor-inducible 14 (Fn14), which is highly expressed in high-grade glioma. The authors demonstrated specific targeting of glioma cells with retained ability to navigate the ECM in rat brain tissue and in a U87 orthotopic xenograft glioma mouse model using CED [249].

This combination of surface modifications, including targeting antibodies and peptides, is frequently used to enhance drug delivery specifically to the targeted tumor cell. While in solid tumors outside the CNS, NPs provide passive accumulation through the enhanced permeability and retention (EPR) effect (i.e., preferential tumor accumulation of nanosized particles through leaky vessels ensuing tumor-induced neoangiogenesis and accompanying inflammatory response), it is unclear whether this concept is directly translatable to brain tumors due to the unique characteristics of the BBB [250,251]. Even though passive accumulation might still occur to a certain degree through tumor-induced BBB disruption, tumor-specific targeting moieties have been used across NP classes to increase brain tumor cell delivery [185,252,253,254].

The most intensely investigated targets for drug delivery to high-grade gliomas are vascular endothelial growth factor (VEGF) and epidermal growth factor receptor (EGFR), including the truncated, constitutively active variant EGFRvIII [67,106,255,256]. Other commonly investigated moieties are TfR-ligands, as discussed above, and chlorotoxin, targeting a chloride ion channel and matrix metalloprotease 2, which have all been shown to be overexpressed in different neuroectodermal tumors [187,206,207,208,209,210,257,258]. Several of these ligands have been successfully conjugated to drug-loaded NPs and demonstrated to increase cytotoxicity and tumor cell selectivity in vitro and CNS accumulation in vivo in orthotopic xenograft glioma mouse models [67,106,229,255,256,258]. However, studies have reported the reliance on the overexpression of the target receptor in the used tumor models [67,106].

Notwithstanding the promising preclinical data, the lack of successful clinical translation highlights the inherent limitations of targeting specific receptors due to inter- and intratumoral heterogeneity, expression changes upon treatment, and the generation of alternative oncogenic mutations, which all promote the development of treatment resistance [53,259,260].

## 4. Novel Strategies and Future Directions

**Figure 4:**
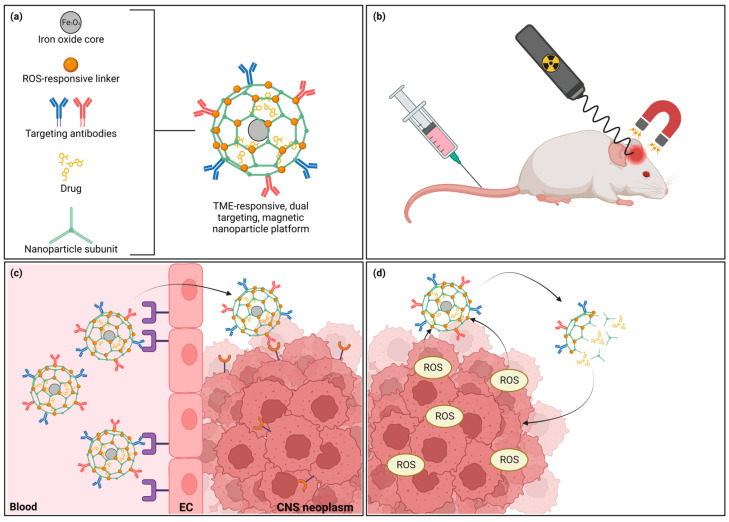
Novel strategies in NP-mediated drug delivery. (**a**) Complex NPs have been constructed, combining magnetic properties, tumor microenvironment (TME)-responsive elements, and multiple targeting strategies into one NP platform for drug delivery. (**b**) After injection, magnetic NPs can increase targeting efficiency using magnetic convection-enhanced diffusion to the region of interest. Furthermore, magnetothermal and radiosensitizing properties allow for additional therapeutic benefits. (**c**) Dual-targeting strategies for recognition of both the blood–brain barrier and brain tumor cells are commonly implemented. (**d**) TME-responsive elements can increase site-directed delivery by allowing cargo release when encountering specific molecules abundant in the TME, e.g., reactive oxygen species (ROS). Abbreviations: NP = nanoparticle, EC = endothelial cell, CNS = central nervous system. Created with BioRender.com.

Notwithstanding the aforementioned strategies to specifically design CNS-targeted NPs with promising preclinical data, no successful clinical translation has been achieved. However, novel technologies are under investigation to further improve NP-based brain tumor therapy by combining several treatment modalities and defining new therapeutic targets (Figure 4).

While many NPs mainly focus on the efficient, targeted delivery of drugs, magnetic NPs, e.g., iron oxide (loaded) NPs, exhibit unique properties, allowing additional therapeutic benefits [261]. Using external magnetic fields, brain targeting can be improved using magnetic convection-enhanced diffusion, usually in combination with regular tumor-targeting ligands [262,263,264,265]. Furthermore, magnetic NPs induce local magnetic hyperthermia when exposed to alternating magnetic fields, providing a noninvasive method to impart local cell death, and act as a radiosensitizer, potentiating the effect of concomitant radiotherapy [266,267]. Similarly, AuNPs display a photothermal effect, providing the possibility of local hyperthermia induction using near-infrared light, while also being a potent radiosensitizer [268,269,270]. As such, the potential of these NPs to potentiate radiotherapy efficacy, improve chemotherapy delivery, and simultaneously allow additional local hyperthermal therapy is offering perspectives to reinforce the current treatment regimens.

The emerging appreciation of the tumor microenvironment (TME) allows ample opportunities for novel NP-based therapies, providing new therapeutic targets and creating new possibilities for TME-responsive NPs to improve site-specific delivery. For example, Hsieh et al. produced a CNS-targeted NP delivering small interfering RNA (siRNA) to silence PD-L1 expression in a GBM mouse model, increasing cytotoxic T cell infiltration and suppressing tumor progression [271]. Furthermore, several groups have created reactive oxygen species (ROS)-responsive NPs, which release their cargo when encountering high ROS concentrations as present in the GBM TME [272,273,274]. Seeing that novel adoptive cellular therapies are currently limited by the immunosuppressive TME and cell-mediated NP delivery has been successful preclinically, combination regimens of adoptive cellular therapies with NP-based TME modulation are under intense investigation [275,276]. Chang et al. introduced MSN loaded with the hypoxia-activated prodrug tirapazamine into anti-GBM chimeric antigen receptor (CAR) neutrophils. In a mouse model, the CAR neutrophils effectively delivered the MSN to the tumor, significantly inhibiting tumor growth and prolonging survival through the combined effect of the CAR neutrophils and the local drug delivery [277]. Furthermore, in solid tumors outside the CNS, pretreatment with TME-modulating NPs or NP-mediated photothermal therapy have also shown promising results, although this has yet to be evaluated in CNS tumors [278,279].

Increasingly, highly complex NPs are being engineered, combining multiple NP types, multiple targeting strategies and various treatment modalities in order to surmount the different biological barriers [226,265,272]. Zhang et al. produced a nanocapsule loaded with anti-VEGFR2 antibodies (inhibiting angiogenesis) crosslinked to anti-CPT1C siRNA (an essential protein for fatty acid oxidation) by a ROS-responsive disulfide crosslinker. The surface was decorated with 2-Deoxy-D-Glucose, a glycolysis inhibitor that is also a substrate for GLUT1, allowing for TMT over the BBB and targeting to the tumor cells. Upon encountering ROS in the TME, the anti-VEGFR2 antibodies, CPT1C siRNA and 2-Deoxy-D-glucose are released, inhibiting angiogenesis, fatty acid oxidation and glycolysis pathways, killing the tumor cells by effectively blocking their energy supply [272]. Another example was recently published by Li et al., combining angiopep-2-decorated EVs, targeting the LRP-1 receptor, with a magnetic NP consisting of an iron oxide core surrounded by a mesoporous silica shell, allowing for both ligand-mediated and magnetic targeting. The EVs were loaded with GPX4 siRNA and the mesoporous silica shell decorated with a dihydroorotate dehydrogenase inhibitor, targeting two important ferroptosis defense pathways, inducing cell death through their combined effect [265]. Similarly, Zhang et al. produced a CNS targeted, MSC membrane-coated, pH-responsive, cupper-based NP loaded with siRNA to induce cuproptosis, a recently uncovered form of cell death [226]. Considering the complexity of these NP platforms reflects the diversity of biological barriers that has to be surmounted, continued efforts are needed in order to achieve effective NP-based treatment strategies for CNS neoplasms.

## 5. Summary and Conclusions

To date, the therapeutic impact of advances in our knowledge of CNS tumors has been significantly hindered by the unique biology which surrounds these tumors, namely the blood–brain barrier, which not only prevents the entry of the vast majority of therapeutics, but actively removes them from the CNS space via the activity of efflux transporters. To overcome the vexing challenges posed by the BBB and increase the CNS tumor therapeutic exposure time, a variety of strategies utilizing nanoparticles have been developed, which enable greater delivery and retention of therapeutics at the site of disease. One such strategy in the CNS targeting nanoparticle space entails the modification of therapeutic-containing nanoparticles with groups which will induce nanoparticle transport into the CNS via transcytosis. To achieve this, nanoparticles are modified with ligands for receptors highly expressed on CNS endothelial cells which when bound will induce transcytosis. Alternatively, the nanoparticle can be modified with a substrate for a transporter highly expressed on CNS endothelial cells which, when bound, similarly induces transcytosis. Ideally, these receptors/transporters are both highly expressed on the CNS endothelial cells and on the tumor itself, as is the case for transferrin and GLUT1, respectively. Another approach for facilitating nanoparticle BBB circumvention involves hitching a ride with cells which are already able to enter the CNS, and which have innate tumor-tropic active homing ability. Examples of this approach have utilized MSCs and various WBCs, and although—to our knowledge—this strategy has yet to be tested in an animal model with an intact BBB, enhanced accumulation of NPs in the tumor region of orthotopic xenograft glioma mouse models has been demonstrated, indicating significant promise for the approach.

In addition to strategies which solely rely on the nanoparticle to bypass the BBB, strategies have been devised which make use of nanoparticles in combination with unique delivery methods which are designed to disrupt the BBB or bypass it entirely. These approaches include intranasal delivery, convection-enhanced delivery, and focused ultrasound. The first two delivery methods are intended to bypass the BBB, while the third attempts to temporarily disrupt the BBB, allowing therapeutics to reach the site of disease. By combining these delivery approaches with nanoparticle formulations which have an enhanced volume of distribution and extended therapeutics release profile, the hope is that the retention of therapeutics at the site of disease can be increased, and, thus, therapeutic efficacy can be achieved. These combination approaches also have the benefit of overcoming one of the major hurdles in the nanoparticle therapeutics space, namely, rapid clearance from the bloodstream by the RES. By directly delivering nanoparticles to the site of disease, this problem of rapid clearance can be ameliorated. Alternatively, the modification of nanoparticles with PEG has been shown to increase circulation time by helping the nanoparticles evade the RES. It is, however, unclear how PEGylation impacts BBB penetration ability, with some studies indicating enhanced penetrance and others indicating diminished BBB penetration. It has also been shown that CD47 expression on nanoparticles can prevent phagocytes from clearing the nanoparticles. Although in its infancy, this strategy of modifying nanoparticles with antiphagocytosis signals holds the promise of helping to defeat rapid nanoparticle clearance by the RES. Increasingly, complex nanoparticles combining several of these strategies and/or exhibiting additional magnetothermal, photothermal, or radiosensitizing effects are being evaluated and are combined with other treatment modalities. In this review, we have covered the current state of the CNS-tumor-targeting nanoparticle space, highlighting the breadth of nanoparticle types being investigated for this use, the strategies being employed to circumvent the BBB, and some of the recent advances in combining nanoparticles with unique delivery methods to overcome the myriad challenges posed by the unique biology surrounding CNS tumors. Taken together, there is significant merit in the continued investigation and development of nanoparticles as therapeutic delivery vehicles for the treatment of CNS tumors in order to translate the successful preclinical investigations into the clinic.

## Figures and Tables

**Table 1:** General strengths and weaknesses of nanoparticle classes.

Nanoparticle Class	Strengths	Weaknesses	References
Lipid-based NP	Simplicity of manufacturing process	Rapid elimination from bloodstream	[58,59,60,61,62,63,64]
	Payload flexibility	CARPA	
	Potential for surface modification		
	Biocompatibility		
Polymeric NP	Precise control over physicochemical properties and drug release profile	Rapid elimination from bloodstream	[65,66,67,68,69,70,71]
	Payload flexibility	Relatively low drug loading capacity	
	Potential for surface modification		
Inorganic NP	Variability in sizes, shapes, and constructs	Low solubility, aggregation	[72,73,74,75,76]
	Unique magnetic and/or photothermal properties, allowing theragnostic applications	Toxicity concerns	
Biological NP	Biocompatibility	Rapid elimination from bloodstream	[61,77,78,79,80,81]
	Inherently functionalized membrane	Low production scalability	
	Payload flexibility	More complex drug loading process	
		Low drug loading capacity	

Abbreviations: NP = nanoparticle, CARPA = complement activation-related pseudoallergy.

## Data Availability

No new data were created or analyzed in this study. Data sharing is not applicable to this article.

## References

[1] Ostrom Q.T., Cioffi G., Waite K., Kruchko C., Barnholtz-Sloan J.S. (2021). CBTRUS Statistical Report: Primary Brain and Other Central Nervous System Tumors Diagnosed in the United States in 2014–2018. Neuro Oncol..

[2] Girardi F., Matz M., Stiller C., You H., Marcos Gragera R., Valkov M.Y., Bulliard J.-L., De P., Morrison D., Wanner M. (2023). Global survival trends for brain tumors, by histology: Analysis of individual records for 556,237 adults diagnosed in 59 countries during 2000–2014 (CONCORD-3). Neuro Oncol..

[3] Gittleman H., Boscia A., Ostrom Q.T., Truitt G., Fritz Y., Kruchko C., Barnholtz-Sloan J.S. (2018). Survivorship in adults with malignant brain and other central nervous system tumor from 2000–2014. Neuro Oncol..

[4] Ostrom Q.T., Price M., Ryan K., Edelson J., Neff C., Cioffi G., Waite K.A., Kruchko C., Barnholtz-Sloan J.S. (2022). CBTRUS Statistical Report: Pediatric Brain Tumor Foundation Childhood and Adolescent Primary Brain and Other Central Nervous System Tumors Diagnosed in the United States in 2014–2018. Neuro Oncol..

[5] Girardi F., Di Carlo V., Stiller C., Gatta G., Woods R.R., Visser O., Lacour B., Tucker T.C., Coleman M.P., Allemani C. (2023). Global survival trends for brain tumors, by histology: Analysis of individual records for 67,776 children diagnosed in 61 countries during 2000–2014 (CONCORD-3). Neuro Oncol..

[6] Warren K.E. (2012). Diffuse intrinsic pontine glioma: Poised for progress. Front. Oncol..

[7] Pollack I.F., Agnihotri S., Broniscer A. (2019). Childhood brain tumors: Current management, biological insights, and future directions. J. Neurosurg. Pediatr..

[8] Schaff L.R., Mellinghoff I.K. (2023). Glioblastoma and Other Primary Brain Malignancies in Adults: A Review. JAMA.

[9] Louis D.N., Perry A., Wesseling P., Brat D.J., Cree I.A., Figarella-Branger D., Hawkins C., Ng H.K., Pfister S.M., Reifenberger G. (2021). The 2021 WHO Classification of Tumors of the Central Nervous System: A summary. Neuro Oncol..

[10] Wen P.Y., Weller M., Lee E.Q., Alexander B.M., Barnholtz-Sloan J.S., Barthel F.P., Batchelor T.T., Bindra R.S., Chang S.M., Chiocca E.A. (2020). Glioblastoma in adults: A Society for Neuro Oncol. (SNO) and European Society of Neuro-Oncology (EANO) consensus review on current management and future directions. Neuro Oncol..

[11] Stupp R., Mason W.P., van den Bent M.J., Weller M., Fisher B., Taphoorn M.J., Belanger K., Brandes A.A., Marosi C., Bogdahn U. (2005). Radiotherapy plus concomitant and adjuvant temozolomide for glioblastoma. N. Engl. J. Med..

[12] Fang F.Y., Rosenblum J.S., Ho W.S., Heiss J.D. (2022). New Developments in the Pathogenesis, Therapeutic Targeting, and Treatment of Pediatric Medulloblastoma. Cancers.

[13] Mendes M., Sousa J.J., Pais A., Vitorino C. (2018). Targeted Theranostic Nanoparticles for Brain Tumor Treatment. Pharmaceutics.

[14] Bellavance M.A., Blanchette M., Fortin D. (2008). Recent advances in blood-brain barrier disruption as a CNS delivery strategy. AAPS J..

[15] Haumann R., Videira J.C., Kaspers G.J.L., van Vuurden D.G., Hulleman E. (2020). Overview of Current Drug Delivery Methods Across the Blood-Brain Barrier for the Treatment of Primary Brain Tumors. CNS Drugs.

[16] Oberoi R.K., Parrish K.E., Sio T.T., Mittapalli R.K., Elmquist W.F., Sarkaria J.N. (2016). Strategies to improve delivery of anticancer drugs across the blood-brain barrier to treat glioblastoma. Neuro Oncol..

[17] Daneman R., Prat A. (2015). The blood-brain barrier. Cold Spring Harb. Perspect. Biol..

[18] Obermeier B., Daneman R., Ransohoff R.M. (2013). Development, maintenance and disruption of the blood-brain barrier. Nat. Med..

[19] Pardridge W.M. (2005). The blood-brain barrier: Bottleneck in brain drug development. NeuroRX.

[20] Abbott N.J., Patabendige A.A., Dolman D.E., Yusof S.R., Begley D.J. (2010). Structure and function of the blood-brain barrier. Neurobiol. Dis..

[21] Pardridge W.M. (2020). The Isolated Brain Microvessel: A Versatile Experimental Model of the Blood-Brain Barrier. Front. Physiol..

[22] Abbott N.J., Rönnbäck L., Hansson E. (2006). Astrocyte-endothelial interactions at the blood-brain barrier. Nat. Rev. Neurosci..

[23] Liu L.R., Liu J.C., Bao J.S., Bai Q.Q., Wang G.Q. (2020). Interaction of Microglia and Astrocytes in the Neurovascular Unit. Front. Immunol..

[24] Xu L., Nirwane A., Yao Y. (2019). Basement membrane and blood-brain barrier. Stroke Vasc. Neurol..

[25] Uemura M.T., Maki T., Ihara M., Lee V.M.Y., Trojanowski J.Q. (2020). Brain Microvascular Pericytes in Vascular Cognitive Impairment and Dementia. Front. Aging Neurosci..

[26] Ballabh P., Braun A., Nedergaard M. (2004). The blood-brain barrier: An overview: Structure, regulation, and clinical implications. Neurobiol. Dis..

[27] Jia W., Lu R., Martin T.A., Jiang W.G. (2014). The role of claudin-5 in blood-brain barrier (BBB) and brain metastases (review). Mol. Med. Rep..

[28] Lochhead J.J., Yang J., Ronaldson P.T., Davis T.P. (2020). Structure, Function, and Regulation of the Blood-Brain Barrier Tight Junction in Central Nervous System Disorders. Front. Physiol..

[29] Tamai I., Tsuji A. (2000). Transporter-mediated permeation of drugs across the blood-brain barrier. J. Pharm. Sci..

[30] Harilal S., Jose J., Parambi D.G.T., Kumar R., Unnikrishnan M.K., Uddin M.S., Mathew G.E., Pratap R., Marathakam A., Mathew B. (2020). Revisiting the blood-brain barrier: A hard nut to crack in the transportation of drug molecules. Brain Res. Bull..

[31] Sun H., Dai H., Shaik N., Elmquist W.F. (2003). Drug efflux transporters in the CNS. Adv. Drug Deliv. Rev..

[32] Löscher W., Potschka H. (2005). Blood-brain barrier active efflux transporters: ATP-binding cassette gene family. NeuroRx.

[33] Singleton W.G.B., Bienemann A.S., Woolley M., Johnson D., Lewis O., Wyatt M.J., Damment S.J.P., Boulter L.J., Killick-Cole C.L., Asby D.J. (2018). The distribution, clearance, and brainstem toxicity of panobinostat administered by convection-enhanced delivery. J. Neurosurg. Pediatr. PED.

[34] Chaves C., Declèves X., Taghi M., Menet M.-C., Lacombe J., Varlet P., Olaciregui N.G., Carcaboso A.M., Cisternino S. (2020). Characterization of the Blood–Brain Barrier Integrity and the Brain Transport of SN-38 in an Orthotopic Xenograft Rat Model of Diffuse Intrinsic Pontine Glioma. Pharmaceutics.

[35] Miklja Z., Yadav V.N., Cartaxo R.T., Siada R., Thomas C.C., Cummings J.R., Mullan B., Stallard S., Paul A., Bruzek A.K. (2020). Everolimus improves the efficacy of dasatinib in PDGFRα-driven glioma. J. Clin. Investig..

[36] Oh J.H., Power E.A., Zhang W., Daniels D.J., Elmquist W.F. (2022). Murine Central Nervous System and Bone Marrow Distribution of the Aurora A Kinase Inhibitor Alisertib: Pharmacokinetics and Exposure at the Sites of Efficacy and Toxicity. J. Pharmacol. Exp. Ther..

[37] Laramy J.K., Kim M., Parrish K.E., Sarkaria J.N., Elmquist W.F. (2018). Pharmacokinetic Assessment of Cooperative Efflux of the Multitargeted Kinase Inhibitor Ponatinib Across the Blood-Brain Barrier. J. Pharmacol. Exp. Ther..

[38] Warren K.E. (2018). Beyond the Blood:Brain Barrier: The Importance of Central Nervous System (CNS) Pharmacokinetics for the Treatment of CNS Tumors, Including Diffuse Intrinsic Pontine Glioma. Front. Oncol..

[39] Bhowmik A., Khan R., Ghosh M.K. (2015). Blood brain barrier: A challenge for effectual therapy of brain tumors. Biomed. Res. Int..

[40] Banks W.A. (2016). From blood–brain barrier to blood–brain interface: New opportunities for CNS drug delivery. Nat. Rev. Drug Discov..

[41] Rathi S., Griffith J.I., Zhang W., Zhang W., Oh J.H., Talele S., Sarkaria J.N., Elmquist W.F. (2022). The influence of the blood-brain barrier in the treatment of brain tumours. J. Intern. Med..

[42] Varlet P., Le Teuff G., Le Deley M.C., Giangaspero F., Haberler C., Jacques T.S., Figarella-Branger D., Pietsch T., Andreiuolo F., Deroulers C. (2020). WHO grade has no prognostic value in the pediatric high-grade glioma included in the HERBY trial. Neuro Oncol..

[43] Sarkaria J.N., Hu L.S., Parney I.F., Pafundi D.H., Brinkmann D.H., Laack N.N., Giannini C., Burns T.C., Kizilbash S.H., Laramy J.K. (2018). Is the blood-brain barrier really disrupted in all glioblastomas? A critical assessment of existing clinical data. Neuro Oncol..

[44] Pafundi D.H., Laack N.N., Youland R.S., Parney I.F., Lowe V.J., Giannini C., Kemp B.J., Grams M.P., Morris J.M., Hoover J.M. (2013). Biopsy validation of 18F-DOPA PET and biodistribution in gliomas for neurosurgical planning and radiotherapy target delineation: Results of a prospective pilot study. Neuro Oncol..

[45] Himes B.T., Zhang L., Daniels D.J. (2019). Treatment Strategies in Diffuse Midline Gliomas With the H3K27M Mutation: The Role of Convection-Enhanced Delivery in Overcoming Anatomic Challenges. Front. Oncol..

[46] Ek C.J., Wong A., Liddelow S.A., Johansson P.A., Dziegielewska K.M., Saunders N.R. (2010). Efflux mechanisms at the developing brain barriers: ABC-transporters in the fetal and postnatal rat. Toxicol. Lett..

[47] Saunders N.R., Liddelow S.A., Dziegielewska K.M. (2012). Barrier mechanisms in the developing brain. Front. Pharmacol..

[48] Verscheijden L.F.M., van Hattem A.C., Pertijs J., de Jongh C.A., Verdijk R.M., Smeets B., Koenderink J.B., Russel F.G.M., de Wildt S.N. (2020). Developmental patterns in human blood-brain barrier and blood-cerebrospinal fluid barrier ABC drug transporter expression. Histochem. Cell Biol..

[49] Mazumder S., Dewangan A.K., Pavurala N. (2017). Enhanced dissolution of poorly soluble antiviral drugs from nanoparticles of cellulose acetate based solid dispersion matrices. Asian J. Pharm. Sci..

[50] Caraway C.A., Gaitsch H., Wicks E.E., Kalluri A., Kunadi N., Tyler B.M. (2022). Polymeric Nanoparticles in Brain Cancer Therapy: A Review of Current Approaches. Polymers.

[51] Anselmo A.C., Mitragotri S. (2019). Nanoparticles in the clinic: An update. Bioeng. Transl. Med..

[52] Genovesi L.A., Puttick S., Millar A., Kojic M., Ji P., Lagendijk A.K., Brighi C., Bonder C.S., Adolphe C., Wainwright B.J. (2021). Patient-derived orthotopic xenograft models of medulloblastoma lack a functional blood-brain barrier. Neuro Oncol..

[53] Becker A., Sells B., Haque S., Chakravarti A. (2021). Tumor Heterogeneity in Glioblastomas: From Light Microscopy to Molecular Pathology. Cancers.

[54] Ruan S., Zhou Y., Jiang X., Gao H. (2021). Rethinking CRITID Procedure of Brain Targeting Drug Delivery: Circulation, Blood Brain Barrier Recognition, Intracellular Transport, Diseased Cell Targeting, Internalization, and Drug Release. Adv. Sci..

[55] Niu X., Chen J., Gao J. (2019). Nanocarriers as a powerful vehicle to overcome blood-brain barrier in treating neurodegenerative diseases: Focus on recent advances. Asian J. Pharm. Sci..

[56] Dong X. (2018). Current Strategies for Brain Drug Delivery. Theranostics.

[57] Rueda F., Cruz L.J. (2017). Targeting the Brain with Nanomedicine. Curr. Pharm. Des..

[58] Koog L., Gandek T.B., Nagelkerke A. (2022). Liposomes and Extracellular Vesicles as Drug Delivery Systems: A Comparison of Composition, Pharmacokinetics, and Functionalization. Adv. Healthc. Mater..

[59] Eloy J.O., Petrilli R., Trevizan L.N.F., Chorilli M. (2017). Immunoliposomes: A review on functionalization strategies and targets for drug delivery. Colloids Surf. B Biointerfaces.

[60] Suk J.S., Xu Q., Kim N., Hanes J., Ensign L.M. (2016). PEGylation as a strategy for improving nanoparticle-based drug and gene delivery. Adv. Drug Deliv. Rev..

[61] Smyth T., Kullberg M., Malik N., Smith-Jones P., Graner M.W., Anchordoquy T.J. (2015). Biodistribution and delivery efficiency of unmodified tumor-derived exosomes. J. Control Release.

[62] Szebeni J., Bedőcs P., Rozsnyay Z., Weiszhár Z., Urbanics R., Rosivall L., Cohen R., Garbuzenko O., Báthori G., Tóth M. (2012). Liposome-induced complement activation and related cardiopulmonary distress in pigs: Factors promoting reactogenicity of Doxil and AmBisome. Nanomed. Nanotechnol. Biol. Med..

[63] Kowalski P.S., Rudra A., Miao L., Anderson D.G. (2019). Delivering the Messenger: Advances in Technologies for Therapeutic mRNA Delivery. Mol. Ther..

[64] Hou X., Zaks T., Langer R., Dong Y. (2021). Lipid nanoparticles for mRNA delivery. Nat. Rev. Mater..

[65] Makadia H.K., Siegel S.J. (2011). Poly Lactic-co-Glycolic Acid (PLGA) as Biodegradable Controlled Drug Delivery Carrier. Polymers.

[66] Song E., Gaudin A., King A.R., Seo Y.E., Suh H.W., Deng Y., Cui J., Tietjen G.T., Huttner A., Saltzman W.M. (2017). Surface chemistry governs cellular tropism of nanoparticles in the brain. Nat. Commun..

[67] Banstola A., Duwa R., Emami F., Jeong J.-H., Yook S. (2020). Enhanced Caspase-Mediated Abrogation of Autophagy by Temozolomide-Loaded and Panitumumab-Conjugated Poly(lactic-co-glycolic acid) Nanoparticles in Epidermal Growth Factor Receptor Overexpressing Glioblastoma Cells. Mol. Pharm..

[68] Jackson C.L., Chanzy H.D., Booy F.P., Drake B.J., Tomalia D.A., Bauer B.J., Amis E.J. (1998). Visualization of Dendrimer Molecules by Transmission Electron Microscopy (TEM):  Staining Methods and Cryo-TEM of Vitrified Solutions. Macromolecules.

[69] Zielińska A., Carreiró F., Oliveira A.M., Neves A., Pires B., Venkatesh D.N., Durazzo A., Lucarini M., Eder P., Silva A.M. (2020). Polymeric Nanoparticles: Production, Characterization, Toxicology and Ecotoxicology. Molecules.

[70] Lim J.-M., Cai T., Mandaric S., Chopra S., Han H., Jang S., Il Choi W., Langer R., Farokhzad O.C., Karnik R. (2019). Drug loading augmentation in polymeric nanoparticles using a coaxial turbulent jet mixer: Yong investigator perspective. J. Colloid Interface Sci..

[71] Gref R., Minamitake Y., Peracchia M.T., Trubetskoy V., Torchilin V., Langer R. (1994). Biodegradable Long-Circulating Polymeric Nanospheres. Science.

[72] Hu X., Zhang Y., Ding T., Liu J., Zhao H. (2020). Multifunctional Gold Nanoparticles: A Novel Nanomaterial for Various Medical Applications and Biological Activities. Front. Bioeng. Biotechnol..

[73] Sababathy M., Ramanathan G., Tan S.C. (2022). Targeted delivery of gold nanoparticles by neural stem cells to glioblastoma for enhanced radiation therapy: A review. AIMS Neurosci..

[74] Arias L., Pessan J., Vieira A., Lima T., Delbem A., Monteiro D. (2018). Iron Oxide Nanoparticles for Biomedical Applications: A Perspective on Synthesis, Drugs, Antimicrobial Activity, and Toxicity. Antibiotics.

[75] Najahi-Missaoui W., Arnold R.D., Cummings B.S. (2020). Safe Nanoparticles: Are We There Yet?. Int. J. Mol. Sci..

[76] Alshehri R., Ilyas A.M., Hasan A., Arnaout A., Ahmed F., Memic A. (2016). Carbon Nanotubes in Biomedical Applications: Factors, Mechanisms, and Remedies of Toxicity. J. Med. Chem..

[77] Hoshino A., Costa-Silva B., Shen T.L., Rodrigues G., Hashimoto A., Tesic Mark M., Molina H., Kohsaka S., Di Giannatale A., Ceder S. (2015). Tumour exosome integrins determine organotropic metastasis. Nature.

[78] Antimisiaris S.G., Mourtas S., Marazioti A. (2018). Exosomes and Exosome-Inspired Vesicles for Targeted Drug Delivery. Pharmaceutics.

[79] Saleh A.F., Lázaro-Ibáñez E., Forsgard M.A.-M., Shatnyeva O., Osteikoetxea X., Karlsson F., Heath N., Ingelsten M., Rose J., Harris J. (2019). Extracellular vesicles induce minimal hepatotoxicity and immunogenicity. Nanoscale.

[80] Zhu X., Badawi M., Pomeroy S., Sutaria D.S., Xie Z., Baek A., Jiang J., Elgamal O.A., Mo X., Perle K.L. (2017). Comprehensive toxicity and immunogenicity studies reveal minimal effects in mice following sustained dosing of extracellular vesicles derived from HEK293T cells. J. Extracell. Vesicles.

[81] Imai T., Takahashi Y., Nishikawa M., Kato K., Morishita M., Yamashita T., Matsumoto A., Charoenviriyakul C., Takakura Y. (2015). Macrophage-dependent clearance of systemically administered B16BL6-derived exosomes from the blood circulation in mice. J. Extracell. Vesicles.

[82] Mondal J., Pillarisetti S., Junnuthula V., Saha M., Hwang S.R., Park I.K., Lee Y.K. (2022). Hybrid exosomes, exosome-like nanovesicles and engineered exosomes for therapeutic applications. J. Control Release.

[83] Gurung S., Perocheau D., Touramanidou L., Baruteau J. (2021). The exosome journey: From biogenesis to uptake and intracellular signalling. Cell Commun. Signal..

[84] Gauro R., Nandave M., Jain V.K., Jain K. (2021). Advances in dendrimer-mediated targeted drug delivery to the brain. J. Nanopart. Res..

[85] Sajid M.I., Jamshaid U., Jamshaid T., Zafar N., Fessi H., Elaissari A. (2016). Carbon nanotubes from synthesis to in vivo biomedical applications. Int. J. Pharm..

[86] Pednekar P.P., Godiyal S.C., Jadhav K.R., Kadam V.J., Ficai A., Grumezescu A.M. (2017). Chapter 23—Mesoporous silica nanoparticles: A promising multifunctional drug delivery system. Nanostructures for Cancer Therapy.

[87] Bharti C., Gulati N., Nagaich U., Pal A. (2015). Mesoporous silica nanoparticles in target drug delivery system: A review. Int. J. Pharm. Investig..

[88] Akbarzadeh A., Rezaei-Sadabady R., Davaran S., Joo S.W., Zarghami N., Hanifehpour Y., Samiei M., Kouhi M., Nejati-Koshki K. (2013). Liposome: Classification, preparation, and applications. Nanoscale Res. Lett..

[89] Gregoriadis G., Ryman B.E. (1972). Fate of Protein-Containing Liposomes Injected into Rats. An Approach to the Treatment of Storage Diseases. Eur. J. Biochem..

[90] Mohamed M., Abu Lila A.S., Shimizu T., Alaaeldin E., Hussein A., Sarhan H.A., Szebeni J., Ishida T. (2019). PEGylated liposomes: Immunological responses. Sci. Technol. Adv. Mater..

[91] Kulkarni J.A., Witzigmann D., Leung J., Tam Y.Y.C., Cullis P.R. (2019). On the role of helper lipids in lipid nanoparticle formulations of siRNA. Nanoscale.

[92] Natarajan J., Baskaran M., Humtsoe L.C., Vadivelan R., Justin A. (2017). Enhanced brain targeting efficacy of Olanzapine through solid lipid nanoparticles. Artif. Cells Nanomed. Biotechnol..

[93] Pashirova T.N., Zueva I.V., Petrov K.A., Babaev V.M., Lukashenko S.S., Rizvanov I.K., Souto E.B., Nikolsky E.E., Zakharova L.Y., Masson P. (2017). Nanoparticle-Delivered 2-PAM for Rat Brain Protection against Paraoxon Central Toxicity. ACS Appl. Mater. Interfaces.

[94] Mitchell M.J., Billingsley M.M., Haley R.M., Wechsler M.E., Peppas N.A., Langer R. (2021). Engineering precision nanoparticles for drug delivery. Nat. Rev. Drug Discov..

[95] Jaiswal M., Dudhe R., Sharma P.K. (2015). Nanoemulsion: An advanced mode of drug delivery system. 3 Biotech.

[96] Carvalho V.F.M., Salata G.C., de Matos J.K.R., Costa-Fernandez S., Chorilli M., Steiner A.A., de Araujo G.L.B., Silveira E.R., Costa-Lotufo L.V., Lopes L.B. (2019). Optimization of composition and obtainment parameters of biocompatible nanoemulsions intended for intraductal administration of piplartine (piperlongumine) and mammary tissue targeting. Int. J. Pharm..

[97] Sánchez-López E., Guerra M., Dias-Ferreira J., Lopez-Machado A., Ettcheto M., Cano A., Espina M., Camins A., Garcia M.L., Souto E.B. (2019). Current Applications of Nanoemulsions in Cancer Therapeutics. Nanomaterials.

[98] Choudhury H., Gorain B., Karmakar S., Biswas E., Dey G., Barik R., Mandal M., Pal T.K. (2014). Improvement of cellular uptake, in vitro antitumor activity and sustained release profile with increased bioavailability from a nanoemulsion platform. Int. J. Pharm..

[99] Gadhave D., Gorain B., Tagalpallewar A., Kokare C. (2019). Intranasal teriflunomide microemulsion: An improved chemotherapeutic approach in glioblastoma. J. Drug Deliv. Sci. Technol..

[100] Shinde R.L., Devarajan P.V. (2017). Docosahexaenoic acid–mediated, targeted and sustained brain delivery of curcumin microemulsion. Drug Deliv..

[101] Bonferoni M., Rossi S., Sandri G., Ferrari F., Gavini E., Rassu G., Giunchedi P. (2019). Nanoemulsions for “Nose-to-Brain” Drug Delivery. Pharmaceutics.

[102] Shieh L., Tamada J., Chen I., Pang J., Domb A., Langer R. (1994). Erosion of a new family of biodegradable polyanhydrides. J. Biomed. Mater. Res..

[103] Tabata Y., Langer R. (1993). Polyanhydride microspheres that display near-constant release of water-soluble model drug compounds. Pharm. Res..

[104] Jain J.P., Modi S., Kumar N. (2008). Hydroxy fatty acid based polyanhydride as drug delivery system: Synthesis, characterization, in vitro degradation, drug release, and biocompatibility. J. Biomed. Mater. Res. A.

[105] Deng Y., Saucier-Sawyer J.K., Hoimes C.J., Zhang J., Seo Y.E., Andrejecsk J.W., Saltzman W.M. (2014). The effect of hyperbranched polyglycerol coatings on drug delivery using degradable polymer nanoparticles. Biomaterials.

[106] Eivazi N., Rahmani R., Paknejad M. (2020). Specific cellular internalization and pH-responsive behavior of doxorubicin loaded PLGA-PEG nanoparticles targeted with anti EGFRvIII antibody. Life Sci..

[107] Gagliardi A., Giuliano E., Venkateswararao E., Fresta M., Bulotta S., Awasthi V., Cosco D. (2021). Biodegradable Polymeric Nanoparticles for Drug Delivery to Solid Tumors. Front. Pharmacol..

[108] Wang W., Meng Q., Li Q., Liu J., Zhou M., Jin Z., Zhao K. (2020). Chitosan Derivatives and Their Application in Biomedicine. Int. J. Mol. Sci..

[109] Cheng J., Teply B., Sherifi I., Sung J., Luther G., Gu F., Levynissenbaum E., Radovicmoreno A., Langer R., Farokhzad O. (2007). Formulation of functionalized PLGA–PEG nanoparticles for in vivo targeted drug delivery. Biomaterials.

[110] Allyn M.M., Luo R.H., Hellwarth E.B., Swindle-Reilly K.E. (2021). Considerations for Polymers Used in Ocular Drug Delivery. Front. Med..

[111] Huntimer L., Ramer-Tait A.E., Petersen L.K., Ross K.A., Walz K.A., Wang C., Hostetter J., Narasimhan B., Wannemuehler M.J. (2013). Evaluation of biocompatibility and administration site reactogenicity of polyanhydride-particle-based platform for vaccine delivery. Adv. Healthc. Mater..

[112] Vela-Ramirez J.E., Goodman J.T., Boggiatto P.M., Roychoudhury R., Pohl N.L.B., Hostetter J.M., Wannemuehler M.J., Narasimhan B. (2015). Safety and Biocompatibility of Carbohydrate-Functionalized Polyanhydride Nanoparticles. AAPS J..

[113] Choi J., Rui Y., Kim J., Gorelick N., Wilson D.R., Kozielski K., Mangraviti A., Sankey E., Brem H., Tyler B. (2020). Nonviral polymeric nanoparticles for gene therapy in pediatric CNS malignancies. Nanomedicine.

[114] Madej M., Kurowska N., Strzalka-Mrozik B. (2022). Polymeric Nanoparticles—Tools in a Drug Delivery System in Selected Cancer Therapies. Appl. Sci..

[115] Wang Y., Hu A. (2014). Carbon quantum dots: Synthesis, properties and applications. J. Mater. Chem. C.

[116] Kushwaha S.K.S., Ghoshal S., Rai A.K., Singh S. (2013). Carbon nanotubes as a novel drug delivery system for anticancer therapy: A review. Braz. J. Pharm. Sci..

[117] Valadi H., Ekström K., Bossios A., Sjöstrand M., Lee J.J., Lötvall J.O. (2007). Exosome-mediated transfer of mRNAs and microRNAs is a novel mechanism of genetic exchange between cells. Nat. Cell Biol..

[118] Peters P.J., Geuze H.J., Van Donk H.A.D., Slot J.W., Griffith J.M., Stam N.J., Clevers H.C., Borst J. (1989). Molecules relevant for T cell-target cell interaction are present in cytolytic granules of human T lymphocytes. Eur. J. Immunol..

[119] van Niel G., Raposo G., Candalh C., Boussac M., Hershberg R., Cerf-Bensussan N., Heyman M. (2001). Intestinal epithelial cells secrete exosome-like vesicles. Gastroenterology.

[120] Raposo G., Nijman H.W., Stoorvogel W., Liejendekker R., Harding C.V., Melief C.J., Geuze H.J. (1996). B lymphocytes secrete antigen-presenting vesicles. J. Exp. Med..

[121] Raposo G., Tenza D., Mecheri S., Peronet R., Bonnerot C., Desaymard C. (1997). Accumulation of Major Histocompatibility Complex Class II Molecules in Mast Cell Secretory Granules and Their Release upon Degranulation. Mol. Biol. Cell.

[122] Wolfers J., Lozier A., Raposo G., Regnault A., Théry C., Masurier C., Flament C., Pouzieux S., Faure F., Tursz T. (2001). Tumor-derived exosomes are a source of shared tumor rejection antigens for CTL cross-priming. Nat. Med..

[123] Zitvogel L., Regnault A., Lozier A., Wolfers J., Flament C., Tenza D., Ricciardi-Castagnoli P., Raposo G., Amigorena S. (1998). Eradication of established murine tumors using a novel cell-free vaccine: Dendritic cell derived exosomes. Nat. Med..

[124] Heijnen H.F., Schiel A.E., Fijnheer R., Geuze H.J., Sixma J.J. (1999). Activated platelets release two types of membrane vesicles: Microvesicles by surface shedding and exosomes derived from exocytosis of multivesicular bodies and alpha-granules. Blood.

[125] Zheng Y., Tu C., Zhang J., Wang J. (2019). Inhibition of multiple myeloma-derived exosomes uptake suppresses the functional response in bone marrow stromal cell. Int. J. Oncol..

[126] Wen S.W., Lima L.G., Lobb R.J., Norris E.L., Hastie M.L., Krumeich S., Moller A. (2019). Breast Cancer-Derived Exosomes Reflect the Cell-of-Origin Phenotype. Proteomics.

[127] Andriolo G., Provasi E., Lo Cicero V., Brambilla A., Soncin S., Torre T., Milano G., Biemmi V., Vassalli G., Turchetto L. (2018). Exosomes From Human Cardiac Progenitor Cells for Therapeutic Applications: Development of a GMP-Grade Manufacturing Method. Front. Physiol..

[128] Yuan D., Zhao Y., Banks W.A., Bullock K.M., Haney M., Batrakova E., Kabanov A.V. (2017). Macrophage exosomes as natural nanocarriers for protein delivery to inflamed brain. Biomaterials.

[129] Yu B., Zhang X., Li X. (2014). Exosomes Derived from Mesenchymal Stem Cells. Int. J. Mol. Sci..

[130] Wan R., Hussain A., Behfar A., Moran S.L., Zhao C. (2022). The Therapeutic Potential of Exosomes in Soft Tissue Repair and Regeneration. Int. J. Mol. Sci..

[131] Liu J., Chen Y., Pei F., Zeng C., Yao Y., Liao W., Zhao Z. (2021). Extracellular Vesicles in Liquid Biopsies: Potential for Disease Diagnosis. BioMed Res. Int..

[132] García-Romero N., Carrión-Navarro J., Esteban-Rubio S., Lázaro-Ibáñez E., Peris-Celda M., Alonso M.M., Guzmán-De-Villoria J., Fernández-Carballal C., De Mendivil A.O., García-Duque S. (2017). DNA sequences within glioma-derived extracellular vesicles can cross the intact blood-brain barrier and be detected in peripheral blood of patients. Oncotarget.

[133] Théry C., Witwer K.W., Aikawa E., Alcaraz M.J., Anderson J.D., Andriantsitohaina R., Antoniou A., Arab T., Archer F., Atkin-Smith G.K. (2018). Minimal information for studies of extracellular vesicles 2018 (MISEV2018): A position statement of the International Society for Extracellular Vesicles and update of the MISEV2014 guidelines. J. Extracell. Vesicles.

[134] Johnstone R.M., Adam M., Hammond J.R., Orr L., Turbide C. (1987). Vesicle formation during reticulocyte maturation. Association of plasma membrane activities with released vesicles (exosomes). J. Biol. Chem..

[135] Men Y., Yelick J., Jin S., Tian Y., Chiang M.S.R., Higashimori H., Brown E., Jarvis R., Yang Y. (2019). Exosome reporter mice reveal the involvement of exosomes in mediating neuron to astroglia communication in the CNS. Nat. Commun..

[136] Nolte-’t Hoen E.N., Buschow S.I., Anderton S.M., Stoorvogel W., Wauben M.H. (2009). Activated T cells recruit exosomes secreted by dendritic cells via LFA-1. Blood.

[137] Zhang B., Wang M., Gong A., Zhang X., Wu X., Zhu Y., Shi H., Wu L., Zhu W., Qian H. (2015). HucMSC-Exosome Mediated-Wnt4 Signaling Is Required for Cutaneous Wound Healing. Stem Cells.

[138] Osaki M., Okada F. (2019). Exosomes and Their Role in Cancer Progression. Yonago Acta Med..

[139] Bang C., Batkai S., Dangwal S., Gupta S.K., Foinquinos A., Holzmann A., Just A., Remke J., Zimmer K., Zeug A. (2014). Cardiac fibroblast–derived microRNA passenger strand-enriched exosomes mediate cardiomyocyte hypertrophy. J. Clin. Investig..

[140] Howitt J., Hill A.F. (2016). Exosomes in the Pathology of Neurodegenerative Diseases. J. Biol. Chem..

[141] Al-Nedawi K., Meehan B., Micallef J., Lhotak V., May L., Guha A., Rak J. (2008). Intercellular transfer of the oncogenic receptor EGFRvIII by microvesicles derived from tumour cells. Nat. Cell Biol..

[142] Thery C., Boussac M., Veron P., Ricciardi-Castagnoli P., Raposo G., Garin J., Amigorena S. (2001). Proteomic analysis of dendritic cell-derived exosomes: A secreted subcellular compartment distinct from apoptotic vesicles. J. Immunol..

[143] Blanchard N., Lankar D., Faure F., Regnault A., Dumont C., Raposo G., Hivroz C. (2002). TCR activation of human T cells induces the production of exosomes bearing the TCR/CD3/zeta complex. J. Immunol..

[144] Clayton A., Court J., Navabi H., Adams M., Mason M.D., Hobot J.A., Newman G.R., Jasani B. (2001). Analysis of antigen presenting cell derived exosomes, based on immuno-magnetic isolation and flow cytometry. J. Immunol. Methods.

[145] Théry C., Zitvogel L., Amigorena S. (2002). Exosomes: Composition, biogenesis and function. Nat. Rev. Immunol..

[146] Skotland T., Sandvig K., Llorente A. (2017). Lipids in exosomes: Current knowledge and the way forward. Prog. Lipid Res..

[147] Théry C., Duban L., Segura E., Véron P., Lantz O., Amigorena S. (2002). Indirect activation of naïve CD4+ T cells by dendritic cell–derived exosomes. Nat. Immunol..

[148] Charoenviriyakul C., Takahashi Y., Morishita M., Nishikawa M., Takakura Y. (2018). Role of Extracellular Vesicle Surface Proteins in the Pharmacokinetics of Extracellular Vesicles. Mol. Pharm..

[149] Huang X., Yuan T., Tschannen M., Sun Z., Jacob H., Du M., Liang M., Dittmar R.L., Liu Y., Liang M. (2013). Characterization of human plasma-derived exosomal RNAs by deep sequencing. BMC Genom..

[150] Kooijmans S.A.A., Aleza C.G., Roffler S.R., Van Solinge W.W., Vader P., Schiffelers R.M. (2016). Display of GPI-anchored anti-EGFR nanobodies on extracellular vesicles promotes tumour cell targeting. J. Extracell. Vesicles.

[151] Kooijmans S.A.A., Fliervoet L.A.L., van der Meel R., Fens M., Heijnen H.F.G., van Bergen En Henegouwen P.M.P., Vader P., Schiffelers R.M. (2016). PEGylated and targeted extracellular vesicles display enhanced cell specificity and circulation time. J. Control Release.

[152] Kamerkar S., Lebleu V.S., Sugimoto H., Yang S., Ruivo C.F., Melo S.A., Lee J.J., Kalluri R. (2017). Exosomes facilitate therapeutic targeting of oncogenic KRAS in pancreatic cancer. Nature.

[153] Murphy D.E., de Jong O.G., Brouwer M., Wood M.J., Lavieu G., Schiffelers R.M., Vader P. (2019). Extracellular vesicle-based therapeutics: Natural versus engineered targeting and trafficking. Exp. Mol. Med..

[154] Lázaro-Ibáñez E., Faruqu F.N., Saleh A.F., Silva A.M., Tzu-Wen Wang J., Rak J., Al-Jamal K.T., Dekker N. (2021). Selection of Fluorescent, Bioluminescent, and Radioactive Tracers to Accurately Reflect Extracellular Vesicle Biodistribution in Vivo. ACS Nano.

[155] Goh W.J., Zou S., Ong W.Y., Torta F., Alexandra A.F., Schiffelers R.M., Storm G., Wang J.-W., Czarny B., Pastorin G. (2017). Bioinspired Cell-Derived Nanovesicles versus Exosomes as Drug Delivery Systems: A Cost-Effective Alternative. Sci. Rep..

[156] Wen Y., Fu Q., Soliwoda A., Zhang S., Zheng M., Mao W., Wan Y. (2022). Cell-derived nanovesicles prepared by membrane extrusion are good substitutes for natural extracellular vesicles. Extracell. Vesicle.

[157] Jang S.C., Kim O.Y., Yoon C.M., Choi D.-S., Roh T.-Y., Park J., Nilsson J., Lötvall J., Kim Y.-K., Gho Y.S. (2013). Bioinspired Exosome-Mimetic Nanovesicles for Targeted Delivery of Chemotherapeutics to Malignant Tumors. ACS Nano.

[158] Goh W.J., Lee C.K., Zou S., Woon E., Czarny B., Pastorin G. (2017). Doxorubicin-loaded cell-derived nanovesicles: An alternative targeted approach for anti-tumor therapy. Int. J. Nanomed..

[159] Wilhelm S., Tavares A.J., Dai Q., Ohta S., Audet J., Dvorak H.F., Chan W.C.W. (2016). Analysis of nanoparticle delivery to tumours. Nat. Rev. Mater..

[160] Sun C., Ding Y., Zhou L., Shi D., Sun L., Webster T.J., Shen Y. (2017). Noninvasive nanoparticle strategies for brain tumor targeting. Nanomedicine.

[161] Li J., Zhao J., Tan T., Liu M., Zeng Z., Zeng Y., Zhang L., Fu C., Chen D., Xie T. (2020). Nanoparticle Drug Delivery System for Glioma and Its Efficacy Improvement Strategies: A Comprehensive Review. Int. J. Nanomed..

[162] Hwang D.W., Choi H., Jang S.C., Yoo M.Y., Park J.Y., Choi N.E., Oh H.J., Ha S., Lee Y.-S., Jeong J.M. (2015). Noninvasive imaging of radiolabeled exosome-mimetic nanovesicle using 99mTc-HMPAO. Sci. Rep..

[163] Klibanov A.L., Maruyama K., Torchilin V.P., Huang L. (1990). Amphipathic Polyethyleneglycols Effectively Prolong the Circulation Time of Liposomes. FEBS Lett..

[164] Hennig R., Pollinger K., Veser A., Breunig M., Goepferich A. (2014). Nanoparticle multivalency counterbalances the ligand affinity loss upon PEGylation. J. Control Release.

[165] Nunes S.S., De Oliveira Silva J., Fernandes R.S., Miranda S.E.M., Leite E.A., De Farias M.A., Portugal R.V., Cassali G.D., Townsend D.M., Oliveira M.C. (2022). PEGylated versus Non-PEGylated pH-Sensitive Liposomes: New Insights from a Comparative Antitumor Activity Study. Pharmaceutics.

[166] Frank T., Klinker F., Falkenburger B.H., Laage R., Lühder F., Göricke B., Schneider A., Neurath H., Desel H., Liebetanz D. (2012). Pegylated granulocyte colony-stimulating factor conveys long-term neuroprotection and improves functional outcome in a model of Parkinson’s disease. Brain.

[167] Elinav E., Niv-Spector L., Katz M., Price T.O., Ali M., Yacobovitz M., Solomon G., Reicher S., Lynch J.L., Halpern Z. (2009). Pegylated leptin antagonist is a potent orexigenic agent: Preparation and mechanism of activity. Endocrinology.

[168] Yang Q., Lai S.K. (2015). Anti-PEG immunity: Emergence, characteristics, and unaddressed questions. Wiley Interdiscip. Rev. Nanomed. Nanobiotechnol..

[169] Chao M.P., Weissman I.L., Majeti R. (2012). The CD47–SIRPα pathway in cancer immune evasion and potential therapeutic implications. Curr. Opin. Immunol..

[170] Belhadj Z., He B., Deng H., Song S., Zhang H., Wang X., Dai W., Zhang Q. (2020). A combined “eat me/don't eat me” strategy based on extracellular vesicles for anticancer nanomedicine. J. Extracell. Vesicles.

[171] Rodriguez P.L., Harada T., Christian D.A., Pantano D.A., Tsai R.K., Discher D.E. (2013). Minimal "Self" Peptides That Inhibit Phagocytic Clearance and Enhance Delivery of Nanoparticles. Science.

[172] Hayat S.M.G., Jaafari M.R., Hatamipour M., Penson P.E., Sahebkar A. (2020). Liposome Circulation Time is Prolonged by CD47 Coating. Protein Pept. Lett..

[173] Yu M., Zheng J. (2015). Clearance Pathways and Tumor Targeting of Imaging Nanoparticles. ACS Nano.

[174] Zhang W., Mehta A., Tong Z., Esser L., Voelcker N.H. (2021). Development of Polymeric Nanoparticles for Blood–Brain Barrier Transfer—Strategies and Challenges. Adv. Sci..

[175] Juliano R.L., Stamp D. (1975). The effect of particle size and charge on the clearance rates of liposomes and liposome encapsulated drugs. Biochem. Biophys. Res. Commun..

[176] Zhang F., Trent Magruder J., Lin Y.-A., Crawford T.C., Grimm J.C., Sciortino C.M., Wilson M.A., Blue M.E., Kannan S., Johnston M.V. (2017). Generation-6 hydroxyl PAMAM dendrimers improve CNS penetration from intravenous administration in a large animal brain injury model. J. Control Release.

[177] Sonavane G., Tomoda K., Makino K. (2008). Biodistribution of colloidal gold nanoparticles after intravenous administration: Effect of particle size. Colloids Surf. B Biointerfaces.

[178] Graham D.K., DeRyckere D., Davies K.D., Earp H.S. (2014). The TAM family: Phosphatidylserine sensing receptor tyrosine kinases gone awry in cancer. Nat. Rev. Cancer.

[179] Matsumoto A., Takahashi Y., Nishikawa M., Sano K., Morishita M., Charoenviriyakul C., Saji H., Takakura Y. (2017). Role of Phosphatidylserine-Derived Negative Surface Charges in the Recognition and Uptake of Intravenously Injected B16BL6-Derived Exosomes by Macrophages. J. Pharm. Sci..

[180] Patel H.M., Tuzel N.S., Ryman B.E. (1983). Inhibitory effect of cholesterol on the uptake of liposomes by liver and spleen. Biochim. Biophys. Acta (BBA)—Gen. Subj..

[181] Champion J.A., Mitragotri S. (2006). Role of target geometry in phagocytosis. Proc. Natl. Acad. Sci. USA.

[182] Huang X., Li L., Liu T., Hao N., Liu H., Chen D., Tang F. (2011). The Shape Effect of Mesoporous Silica Nanoparticles on Biodistribution, Clearance, and Biocompatibility in Vivo. ACS Nano.

[183] Arnida;Janát-Amsbury M.M., Ray A., Peterson C.M., Ghandehari H. (2011). Geometry and surface characteristics of gold nanoparticles influence their biodistribution and uptake by macrophages. Eur. J. Pharm. Biopharm..

[184] Yang T., Martin P., Fogarty B., Brown A., Schurman K., Phipps R., Yin V.P., Lockman P., Bai S. (2015). Exosome Delivered Anticancer Drugs Across the Blood-Brain Barrier for Brain Cancer Therapy in Danio Rerio. Pharm. Res..

[185] Na J.H., Koo H., Lee S., Min K.H., Park K., Yoo H., Lee S.H., Park J.H., Kwon I.C., Jeong S.Y. (2011). Real-time and non-invasive optical imaging of tumor-targeting glycol chitosan nanoparticles in various tumor models. Biomaterials.

[186] Maksimenko O., Malinovskaya J., Shipulo E., Osipova N., Razzhivina V., Arantseva D., Yarovaya O., Mostovaya U., Khalansky A., Fedoseeva V. (2019). Doxorubicin-loaded PLGA nanoparticles for the chemotherapy of glioblastoma: Towards the pharmaceutical development. Int. J. Pharm..

[187] Fang C., Wang K., Stephen Z.R., Mu Q., Kievit F.M., Chiu D.T., Press O.W., Zhang M. (2015). Temozolomide Nanoparticles for Targeted Glioblastoma Therapy. ACS Appl. Mater. Interfaces.

[188] Marchetti L., Engelhardt B. (2020). Immune cell trafficking across the blood-brain barrier in the absence and presence of neuroinflammation. Vasc. Biol..

[189] Liu L., Eckert M.A., Riazifar H., Kang D.-K., Agalliu D., Zhao W. (2013). From Blood to the Brain: Can Systemically Transplanted Mesenchymal Stem Cells Cross the Blood-Brain Barrier?. Stem Cells Int..

[190] Cao M., Mao J., Duan X., Lu L., Zhang F., Lin B., Chen M., Zheng C., Zhang X., Shen J. (2018). In vivo tracking of the tropism of mesenchymal stem cells to malignant gliomas using reporter gene-based MR imaging. Int. J. Cancer.

[191] Simionescu M., Ghinea N., Fixman A., Lasser M., Kukes L., Simionescu N., Palade G.E. (1988). The cerebral microvasculature of the rat: Structure and luminal surface properties during early development. J. Submicrosc. Cytol. Pathol..

[192] Azarmi M., Maleki H., Nikkam N., Malekinejad H. (2020). Transcellular brain drug delivery: A review on recent advancements. Int. J. Pharm..

[193] Moura R.P., Martins C., Pinto S., Sousa F., Sarmento B. (2019). Blood-brain barrier receptors and transporters: An insight on their function and how to exploit them through nanotechnology. Expert Opin. Drug Deliv..

[194] Moscariello P., Ng D.Y.W., Jansen M., Weil T., Luhmann H.J., Hedrich J. (2018). Brain Delivery of Multifunctional Dendrimer Protein Bioconjugates. Adv. Sci..

[195] Albertazzi L., Serresi M., Albanese A., Beltram F. (2010). Dendrimer Internalization and Intracellular Trafficking in Living Cells. Mol. Pharm..

[196] Ordóñez-Gutiérrez L., Re F., Bereczki E., Ioja E., Gregori M., Andersen A.J., Antón M., Moghimi S.M., Pei J.-J., Masserini M. (2015). Repeated intraperitoneal injections of liposomes containing phosphatidic acid and cardiolipin reduce amyloid-β levels in APP/PS1 transgenic mice. Nanomed. Nanotechnol. Biol. Med..

[197] Dehouck B., Fenart L., Dehouck M.-P., Pierce A., Torpier G., Cecchelli R. (1997). A New Function for the LDL Receptor: Transcytosis of LDL across the Blood–Brain Barrier. J. Cell Biol..

[198] Jefferies W.A., Brandon M.R., Hunt S.V., Williams A.F., Gatter K.C., Mason D.Y. (1984). Transferrin receptor on endothelium of brain capillaries. Nature.

[199] Neves A.R., Queiroz J.F., Lima S.A.C., Reis S. (2017). Apo E-Functionalization of Solid Lipid Nanoparticles Enhances Brain Drug Delivery: Uptake Mechanism and Transport Pathways. Bioconj. Chem..

[200] Hu K., Shi Y., Jiang W., Han J., Huang S., Jiang X. (2011). Lactoferrin conjugated PEG-PLGA nanoparticles for brain delivery: Preparation, characterization and efficacy in Parkinson's disease. Int. J. Pharm..

[201] Shilo M., Motiei M., Hana P., Popovtzer R. (2014). Transport of nanoparticles through the blood-brain barrier for imaging and therapeutic applications. Nanoscale.

[202] Xin H., Sha X., Jiang X., Zhang W., Chen L., Fang X. (2012). Anti-glioblastoma efficacy and safety of paclitaxel-loading Angiopep-conjugated dual targeting PEG-PCL nanoparticles. Biomaterials.

[203] Zhang W., Liu Q.Y., Haqqani A.S., Leclerc S., Liu Z., Fauteux F., Baumann E., Delaney C.E., Ly D., Star A.T. (2020). Differential expression of receptors mediating receptor-mediated transcytosis (RMT) in brain microvessels, brain parenchyma and peripheral tissues of the mouse and the human. Fluids Barriers CNS.

[204] Hawkins B.T., Egleton R.D., Davis T.P. (2005). Modulation of cerebral microvascular permeability by endothelial nicotinic acetylcholine receptors. Am. J. Physiol.-Heart Circ. Physiol..

[205] Ramalho M.J., Sevin E., Gosselet F., Lima J., Coelho M.A.N., Loureiro J.A., Pereira M.C. (2018). Receptor-mediated PLGA nanoparticles for glioblastoma multiforme treatment. Int. J. Pharm..

[206] Kuang Y., Jiang X., Zhang Y., Lu Y., Ma H., Guo Y., Zhang Y., An S., Li J., Liu L. (2016). Dual Functional Peptide-Driven Nanoparticles for Highly Efficient Glioma-Targeting and Drug Codelivery. Mol. Pharm..

[207] Johnsen K.B., Bak M., Melander F., Thomsen M.S., Burkhart A., Kempen P.J., Andresen T.L., Moos T. (2019). Modulating the antibody density changes the uptake and transport at the blood-brain barrier of both transferrin receptor-targeted gold nanoparticles and liposomal cargo. J. Control Release.

[208] Paris-Robidas S., Emond V., Tremblay C., Soulet D., Calon F. (2011). In Vivo Labeling of Brain Capillary Endothelial Cells after Intravenous Injection of Monoclonal Antibodies Targeting the Transferrin Receptor. Mol. Pharmacol..

[209] Mao J., Meng X., Zhao C., Yang Y., Liu G. (2019). Development of transferrin-modified poly(lactic-co-glycolic acid) nanoparticles for glioma therapy. Anti-Cancer Drugs.

[210] Cabral Filho P.E., Cardoso A.L.C., Pereira M.I.A., Ramos A.P.M., Hallwass F., Castro M.M.C.A., Geraldes C.F.G.C., Santos B.S., Pedroso de Lima M.C., Pereira G.A.L. (2016). CdTe quantum dots as fluorescent probes to study transferrin receptors in glioblastoma cells. Biochim. Biophys. Acta (BBA)—Gen. Subj..

[211] Roberts R.L., Fine R.E., Sandra A. (1993). Receptor-mediated endocytosis of transferrin at the blood-brain barrier. J. Cell Sci..

[212] Uchida Y., Ohtsuki S., Katsukura Y., Ikeda C., Suzuki T., Kamiie J., Terasaki T. (2011). Quantitative targeted absolute proteomics of human blood-brain barrier transporters and receptors. J. Neurochem..

[213] Maussang D., Rip J., van Kregten J., van den Heuvel A., van der Pol S., van der Boom B., Reijerkerk A., Chen L., de Boer M., Gaillard P. (2016). Glutathione conjugation dose-dependently increases brain-specific liposomal drug delivery in vitro and in vivo. Drug Discov. Today Technol..

[214] Da Silva-Candal A., Brown T., Krishnan V., Lopez-Loureiro I., Ávila-Gómez P., Pusuluri A., Pérez-Díaz A., Correa-Paz C., Hervella P., Castillo J. (2019). Shape effect in active targeting of nanoparticles to inflamed cerebral endothelium under static and flow conditions. J. Control Release.

[215] Kolhar P., Anselmo A.C., Gupta V., Pant K., Prabhakarpandian B., Ruoslahti E., Mitragotri S. (2013). Using shape effects to target antibody-coated nanoparticles to lung and brain endothelium. Proc. Natl. Acad. Sci. USA.

[216] Nowak M., Brown T.D., Graham A., Helgeson M.E., Mitragotri S. (2020). Size, shape, and flexibility influence nanoparticle transport across brain endothelium under flow. Bioeng. Transl. Med..

[217] Takeshita Y., Ransohoff R.M. (2012). Inflammatory cell trafficking across the blood-brain barrier: Chemokine regulation and in vitro models. Immunol. Rev..

[218] Han Y., Li X., Zhang Y., Han Y., Chang F., Ding J. (2019). Mesenchymal Stem Cells for Regenerative Medicine. Cells.

[219] Roger M., Clavreul A., Venier-Julienne M.C., Passirani C., Sindji L., Schiller P., Montero-Menei C., Menei P. (2010). Mesenchymal stem cells as cellular vehicles for delivery of nanoparticles to brain tumors. Biomaterials.

[220] Li L., Guan Y., Liu H., Hao N., Liu T., Meng X., Fu C., Li Y., Qu Q., Zhang Y. (2011). Silica Nanorattle–Doxorubicin-Anchored Mesenchymal Stem Cells for Tumor-Tropic Therapy. ACS Nano.

[221] Choi M.-R., Stanton-Maxey K.J., Stanley J.K., Levin C.S., Bardhan R., Akin D., Badve S., Sturgis J., Robinson J.P., Bashir R. (2007). A Cellular Trojan Horse for Delivery of Therapeutic Nanoparticles into Tumors. Nano Lett..

[222] Ibarra L.E., Beaugé L., Arias-Ramos N., Rivarola V.A., Chesta C.A., López-Larrubia P., Palacios R.E. (2020). Trojan horse monocyte-mediated delivery of conjugated polymer nanoparticles for improved photodynamic therapy of glioblastoma. Nanomedicine.

[223] Li Z., Huang H., Tang S., Li Y., Yu X.F., Wang H., Li P., Sun Z., Zhang H., Liu C. (2016). Small gold nanorods laden macrophages for enhanced tumor coverage in photothermal therapy. Biomaterials.

[224] Chu D., Gao J., Wang Z. (2015). Neutrophil-Mediated Delivery of Therapeutic Nanoparticles across Blood Vessel Barrier for Treatment of Inflammation and Infection. ACS Nano.

[225] Steinfeld U., Pauli C., Kaltz N., Bergemann C., Lee H.H. (2006). T lymphocytes as potential therapeutic drug carrier for cancer treatment. Int. J. Pharm..

[226] Zhang J., Han M., Zhang J., Abdalla M., Sun P., Yang Z., Zhang C., Liu Y., Chen C., Jiang X. (2023). Syphilis mimetic nanoparticles for cuproptosis-based synergistic cancer therapy via reprogramming copper metabolism. Int. J. Pharm..

[227] Ji J., Lian W., Zhang Y., Lin D., Wang J., Mo Y., Xu X., Hou C., Ma C., Zheng Y. (2023). Preoperative administration of a biomimetic platelet nanodrug enhances postoperative drug delivery by bypassing thrombus. Int. J. Pharm..

[228] Zhuang X., Xiang X., Grizzle W., Sun D., Zhang S., Axtell R.C., Ju S., Mu J., Zhang L., Steinman L. (2011). Treatment of Brain Inflammatory Diseases by Delivering Exosome Encapsulated Anti-inflammatory Drugs from the Nasal Region to the Brain. Mol. Ther..

[229] Sousa F., Dhaliwal H.K., Gattacceca F., Sarmento B., Amiji M.M. (2019). Enhanced anti-angiogenic effects of bevacizumab in glioblastoma treatment upon intranasal administration in polymeric nanoparticles. J. Control Release.

[230] Djupesland P.G. (2013). Nasal drug delivery devices: Characteristics and performance in a clinical perspective—A review. Drug Deliv. Transl. Res..

[231] Bobo R.H., Laske D.W., Akbasak A., Morrison P.F., Dedrick R.L., Oldfield E.H. (1994). Convection-enhanced delivery of macromolecules in the brain. Proc. Natl. Acad. Sci. USA.

[232] Lonser R.R., Sarntinoranont M., Morrison P.F., Oldfield E.H. (2015). Convection-enhanced delivery to the central nervous system. J. Neurosurg..

[233] Zhou Z., Singh R., Souweidane M.M. (2017). Convection-Enhanced Delivery for Diffuse Intrinsic Pontine Glioma Treatment. Curr. Neuropharmacol..

[234] Souweidane M.M., Kramer K., Pandit-Taskar N., Zhou Z., Haque S., Zanzonico P., Carrasquillo J.A., Lyashchenko S.K., Thakur S.B., Donzelli M. (2018). Convection-enhanced delivery for diffuse intrinsic pontine glioma: A single-centre, dose-escalation, phase 1 trial. Lancet Oncol..

[235] Bander E.D., Ramos A.D., Wembacher-Schroeder E., Ivasyk I., Thomson R., Morgenstern P.F., Souweidane M.M. (2020). Repeat convection-enhanced delivery for diffuse intrinsic pontine glioma. J. Neurosurg. Pediatr..

[236] Mueller S., Kline C., Villanueva-Meyer J., Hoffman C., Raber S., Bonner E., Nazarian J., Lundy S., Molinaro A.M., Prados M. (2020). EPCT-12. PNOC015: PHASE 1 STUDY OF MTX110 (AQUEOUS PANOBINOSTAT) DELIVERED BY CONVECTION ENHANCED DELIVERY (CED) IN CHILDREN WITH NEWLY DIAGNOSED DIFFUSE INTRINSIC PONTINE GLIOMA (DIPG) PREVIOUSLY TREATED WITH RADIATION THERAPY. Neuro Oncol..

[237] Zacharoulis S., Szalontay L., CreveCoeur T., Neira J., Higgins D., Englander Z., Spinazzi E., Sethi C., Canoll P., Garvin J. (2022). DDEL-07. A Phase I study examining the feasibility of intermittent convection-enhanced delivery (CED) of MTX110 for the treatment of children with newly diagnosed diffuse midline gliomas (DMGs). Neuro Oncol..

[238] Heiss J.D., Jamshidi A., Shah S., Martin S., Wolters P.L., Argersinger D.P., Warren K.E., Lonser R.R. (2018). Phase I trial of convection-enhanced delivery of IL13-Pseudomonas toxin in children with diffuse intrinsic pontine glioma. J. Neurosurg. Pediatr..

[239] Kunwar S., Chang S., Westphal M., Vogelbaum M., Sampson J., Barnett G., Shaffrey M., Ram Z., Piepmeier J., Prados M. (2010). Phase III randomized trial of CED of IL13-PE38QQR vs Gliadel wafers for recurrent glioblastoma. Neuro Oncol..

[240] Sampson J.H., Archer G., Pedain C., Wembacher-Schröder E., Westphal M., Kunwar S., Vogelbaum M.A., Coan A., Herndon J.E., Raghavan R. (2010). Poor drug distribution as a possible explanation for the results of the PRECISE trial. J. Neurosurg..

[241] Mueller S., Polley M.Y., Lee B., Kunwar S., Pedain C., Wembacher-Schröder E., Mittermeyer S., Westphal M., Sampson J.H., Vogelbaum M.A. (2011). Effect of imaging and catheter characteristics on clinical outcome for patients in the PRECISE study. J. Neurooncol..

[242] Bredlau A.L., Dixit S., Chen C., Broome A.M. (2017). Nanotechnology Applications for Diffuse Intrinsic Pontine Glioma. Curr. Neuropharmacol..

[243] Zhang R., Saito R., Mano Y., Kanamori M., Sonoda Y., Kumabe T., Tominaga T. (2014). Concentration rather than dose defines the local brain toxicity of agents that are effectively distributed by convection-enhanced delivery. J. Neurosci. Methods.

[244] Zacharoulis S., Columbia University CED of MTX110 Newly Diagnosed Diffuse Midline Gliomas. https://ClinicalTrials.gov/show/NCT04264143.

[245] Cheng Z., Zhang J., Liu H., Li Y., Zhao Y., Yang E. (2010). Central Nervous System Penetration for Small Molecule Therapeutic Agents Does Not Increase in Multiple Sclerosis- and Alzheimer’s Disease-Related Animal Models Despite Reported Blood-Brain Barrier Disruption. Drug Metab. Dispos..

[246] Somjen G., Segal M., Herreras O. (1991). Osmotic-hypertensive opening of the blood-brain barrier in rats does not necessarily provide access for potassium to cerebral interstitial fluid. Exp. Physiol..

[247] Nance E., Timbie K., Miller G.W., Song J., Louttit C., Klibanov A.L., Shih T.-Y., Swaminathan G., Tamargo R.J., Woodworth G.F. (2014). Non-invasive delivery of stealth, brain-penetrating nanoparticles across the blood − brain barrier using MRI-guided focused ultrasound. J. Control Release.

[248] Nance E.A., Woodworth G.F., Sailor K.A., Shih T.-Y., Xu Q., Swaminathan G., Xiang D., Eberhart C., Hanes J. (2012). A Dense Poly(Ethylene Glycol) Coating Improves Penetration of Large Polymeric Nanoparticles Within Brain Tissue. Sci. Transl. Med..

[249] Schneider C.S., Perez J.G., Cheng E., Zhang C., Mastorakos P., Hanes J., Winkles J.A., Woodworth G.F., Kim A.J. (2015). Minimizing the non-specific binding of nanoparticles to the brain enables active targeting of Fn14-positive glioblastoma cells. Biomaterials.

[250] Matsumura Y., Maeda H. (1986). A new concept for macromolecular therapeutics in cancer chemotherapy: Mechanism of tumoritropic accumulation of proteins and the antitumor agent smancs. Cancer Res..

[251] Gabizon A., Shmeeda H., Barenholz Y. (2003). Pharmacokinetics of Pegylated Liposomal Doxorubicin. Clin. Pharmacokinet..

[252] Mo F., Pellerino A., Soffietti R., Rudà R. (2021). Blood–Brain Barrier in Brain Tumors: Biology and Clinical Relevance. Int. J. Mol. Sci..

[253] Siegal T., Horowitz A., Gabizon A. (1995). Doxorubicin encapsulated in sterically stabilized liposomes for the treatment of a brain tumor model: Biodistribution and therapeutic efficacy. J. Neurosurg..

[254] Sarin H., Kanevsky A.S., Wu H., Brimacombe K.R., Fung S.H., Sousa A.A., Auh S., Wilson C.M., Sharma K., Aronova M.A. (2008). Effective transvascular delivery of nanoparticles across the blood-brain tumor barrier into malignant glioma cells. J. Transl. Med..

[255] Shein S.A., Kuznetsov I.I., Abakumova T.O., Chelushkin P.S., Melnikov P.A., Korchagina A.A., Bychkov D.A., Seregina I.F., Bolshov M.A., Kabanov A.V. (2016). VEGF- and VEGFR2-Targeted Liposomes for Cisplatin Delivery to Glioma Cells. Mol. Pharm..

[256] Shein S.A., Nukolova N.V., Korchagina A.A., Abakumova T.O., Kiuznetsov I.I., Abakumov M.A., Baklaushev V.P., Gurina O.I., Chekhonin V.P. (2015). Site-Directed Delivery of VEGF-Targeted Liposomes into Intracranial C6 Glioma. Bull. Exp. Biol. Med..

[257] Veiseh M., Gabikian P., Bahrami S.B., Veiseh O., Zhang M., Hackman R.C., Ravanpay A.C., Stroud M.R., Kusuma Y., Hansen S.J. (2007). Tumor Paint: A Chlorotoxin:Cy5.5 Bioconjugate for Intraoperative Visualization of Cancer Foci. Cancer Res..

[258] Mortensen J.H., Jeppesen M., Pilgaard L., Agger R., Duroux M., Zachar V., Moos T. (2013). Targeted Antiepidermal Growth Factor Receptor (Cetuximab) Immunoliposomes Enhance Cellular Uptake In Vitro and Exhibit Increased Accumulation in an Intracranial Model of Glioblastoma Multiforme. J. Drug Deliv..

[259] Greenall S.A., McKenzie M., Seminova E., Dolezal O., Pearce L., Bentley J., Kuchibhotla M., Chen S.C., McDonald K.L., Kornblum H.I. (2019). Most clinical anti-EGFR antibodies do not neutralize both wtEGFR and EGFRvIII activation in glioma. Neuro Oncol..

[260] Pan P.C., Magge R.S. (2020). Mechanisms of EGFR Resistance in Glioblastoma. Int. J. Mol. Sci..

[261] Dhar D., Ghosh S., Das S., Chatterjee J. (2022). A review of recent advances in magnetic nanoparticle-based theranostics of glioblastoma. Nanomedicine.

[262] Ganipineni L.P., Ucakar B., Joudiou N., Bianco J., Danhier P., Zhao M., Bastiancich C., Gallez B., Danhier F., Préat V. (2018). Magnetic targeting of paclitaxel-loaded poly(lactic-co-glycolic acid)-based nanoparticles for the treatment of glioblastoma. Int. J. Nanomed..

[263] Heggannavar G.B., Hiremath C.G., Achari D.D., Pangarkar V.G., Kariduraganavar M.Y. (2018). Development of Doxorubicin-Loaded Magnetic Silica–Pluronic F-127 Nanocarriers Conjugated with Transferrin for Treating Glioblastoma across the Blood–Brain Barrier Using an in Vitro Model. ACS Omega.

[264] Norouzi M., Yathindranath V., Thliveris J.A., Kopec B.M., Siahaan T.J., Miller D.W. (2020). Doxorubicin-loaded iron oxide nanoparticles for glioblastoma therapy: A combinational approach for enhanced delivery of nanoparticles. Sci. Rep..

[265] Li B., Chen X., Qiu W., Zhao R., Duan J., Zhang S., Pan Z., Zhao S., Guo Q., Qi Y. (2022). Synchronous Disintegration of Ferroptosis Defense Axis via Engineered Exosome-Conjugated Magnetic Nanoparticles for Glioblastoma Therapy. Adv. Sci..

[266] Calatayud M.P., Soler E., Torres T.E., Campos-Gonzalez E., Junquera C., Ibarra M.R., Goya G.F. (2017). Cell damage produced by magnetic fluid hyperthermia on microglial BV2 cells. Sci. Rep..

[267] Shen Z., Liu T., Yang Z., Zhou Z., Tang W., Fan W., Liu Y., Mu J., Li L., Bregadze V.I. (2020). Small-sized gadolinium oxide based nanoparticles for high-efficiency theranostics of orthotopic glioblastoma. Biomaterials.

[268] Kefayat A., Ghahremani F., Motaghi H., Amouheidari A. (2019). Ultra-small but ultra-effective: Folic acid-targeted gold nanoclusters for enhancement of intracranial glioma tumors' radiation therapy efficacy. Nanomed. Nanotechnol. Biol. Med..

[269] Goubault C., Jarry U., Bostoen M., Eliat P.A., Kahn M.L., Pedeux R., Guillaudeux T., Gauffre F., Chevance S. (2022). Radiosensitizing Fe-Au nanocapsules (hybridosomes(R)) increase survival of GL261 brain tumor-bearing mice treated by radiotherapy. Nanomedicine.

[270] Jing Z., Li M., Wang H., Yang Z., Zhou S., Ma J., Meng E., Zhang H., Liang W., Hu W. (2021). Gallic acid-gold nanoparticles enhance radiation-induced cell death of human glioma U251 cells. IUBMB Life.

[271] Hsieh H.T., Huang H.C., Chung C.W., Chiang C.C., Hsia T., Wu H.F., Huang R.L., Chiang C.S., Wang J., Lu T.T. (2022). CXCR4-targeted nitric oxide nanoparticles deliver PD-L1 siRNA for immunotherapy against glioblastoma. J. Control Release.

[272] Zhang Y., Ren Y., Xu H., Li L., Qian F., Wang L., Quan A., Ma H., Liu H., Yu R. (2023). Cascade-Responsive 2-DG Nanocapsules Encapsulate aV-siCPT1C Conjugates to Inhibit Glioblastoma through Multiple Inhibition of Energy Metabolism. ACS Appl. Mater. Interfaces.

[273] Rinaldi A., Caraffi R., Grazioli M.V., Oddone N., Giardino L., Tosi G., Vandelli M.A., Calzà L., Ruozi B., Duskey J.T. (2022). Applications of the ROS-Responsive Thioketal Linker for the Production of Smart Nanomedicines. Polymers.

[274] Oddone N., Boury F., Garcion E., Grabrucker A.M., Martinez M.C., Da Ros F., Janaszewska A., Forni F., Vandelli M.A., Tosi G. (2020). Synthesis, Characterization, and In Vitro Studies of an Reactive Oxygen Species (ROS)-Responsive Methoxy Polyethylene Glycol-Thioketal-Melphalan Prodrug for Glioblastoma Treatment. Front. Pharmacol..

[275] Liu Z., Zhou Z., Dang Q., Xu H., Lv J., Li H., Han X. (2022). Immunosuppression in tumor immune microenvironment and its optimization from CAR-T cell therapy. Theranostics.

[276] Balakrishnan P.B., Sweeney E.E. (2021). Nanoparticles for Enhanced Adoptive T Cell Therapies and Future Perspectives for CNS Tumors. Front. Immunol..

[277] Chang Y., Cai X., Syahirah R., Yao Y., Xu Y., Jin G., Bhute V.J., Torregrosa-Allen S., Elzey B.D., Won Y.-Y. (2023). CAR-neutrophil mediated delivery of tumor-microenvironment responsive nanodrugs for glioblastoma chemo-immunotherapy. Nat. Commun..

[278] Zhang F., Stephan S.B., Ene C.I., Smith T.T., Holland E.C., Stephan M.T. (2018). Nanoparticles That Reshape the Tumor Milieu Create a Therapeutic Window for Effective T-cell Therapy in Solid Malignancies. Cancer Res..

[279] Chen Q., Hu Q., Dukhovlinova E., Chen G., Ahn S., Wang C., Ogunnaike E.A., Ligler F.S., Dotti G., Gu Z. (2019). Photothermal Therapy Promotes Tumor Infiltration and Antitumor Activity of CAR T Cells. Adv. Mater..

